# Mapping crossover events of mouse meiotic recombination by restriction fragment ligation-based Refresh-seq

**DOI:** 10.1038/s41421-023-00638-9

**Published:** 2024-03-05

**Authors:** Yan Wang, Yijun Chen, Junpeng Gao, Haoling Xie, Yuqing Guo, Jingwei Yang, Jun’e Liu, Zonggui Chen, Qingqing Li, Mengyao Li, Jie Ren, Lu Wen, Fuchou Tang

**Affiliations:** 1 Biomedical Pioneering Innovation Center, School of Life Sciences, Peking University, Beijing, China; 2 Beijing Advanced Innovation Center for Genomics (ICG), Ministry of Education Key Laboratory of Cell Proliferation and Differentiation, Beijing, China; 3 Peking-Tsinghua Center for Life Sciences, Academy for Advanced Interdisciplinary Studies, Peking University, Beijing, China; 4 Emergency Center, Zhongnan Hospital of Wuhan University, Wuhan, Hubei China; 5 Changping Laboratory, Beijing, China

**Keywords:** Whole genome amplification, Developmental biology, Chromosomes

## Abstract

Single-cell whole-genome sequencing methods have undergone great improvements over the past decade. However, allele dropout, which means the inability to detect both alleles simultaneously in an individual diploid cell, largely restricts the application of these methods particularly for medical applications. Here, we develop a new single-cell whole-genome sequencing method based on third-generation sequencing (TGS) platform named Refresh-seq (restriction fragment ligation-based genome amplification and TGS). It is based on restriction endonuclease cutting and ligation strategy in which two alleles in an individual cell can be cut into equal fragments and tend to be amplified simultaneously. As a new single-cell long-read genome sequencing method, Refresh-seq features much lower allele dropout rate compared with SMOOTH-seq. Furthermore, we apply Refresh-seq to 688 sperm cells and 272 female haploid cells (secondary polar bodies and parthenogenetic oocytes) from F1 hybrid mice. We acquire high-resolution genetic map of mouse meiosis recombination at low sequencing depth and reveal the sexual dimorphism in meiotic crossovers. We also phase the structure variations (deletions and insertions) in sperm cells and female haploid cells with high precision. Refresh-seq shows great performance in screening aneuploid sperm cells and oocytes due to the low allele dropout rate and has great potential for medical applications such as preimplantation genetic diagnosis.

## Introduction

A multicellular organism is composed of individual cells and genetic information exists as individual chromosomes in each cell. Development of single-cell whole-genome sequencing techniques has enabled us to amplify and sequence the diploid genome in a single cell to study the genetic heterogeneities within a population of cells, including single-nucleotide variations (SNVs), copy-number variations (CNVs), and structural variations (SVs)^1^. A variety of single-cell genome sequencing technologies based on the next-generation sequencing (NGS) platforms have been developed, such as degenerate oligonucleotide-primed polymerase chain reaction (DOP-PCR)^2^, multiple displacement amplification (MDA)^3^, multiple annealing and looping-based amplification cycles (MALBAC)^4^, emulsion WGA (eWGA)^5^, linear amplification via transposon insertion (LIANTI)^6^, primary template-directed amplification (PTA)^7^ and multiplexed end-tagging amplification of complementary strands (META-CS)^8^. Although these techniques are powerful in the detection of CNVs and SNVs due to the high accuracy of the NGS platforms, they are limited by the short read-length and thus have poor performance in the detection of SVs. SVs include deletion, insertion, duplication, inversion and translocation, which are important categories of genetic variations underlying many human diseases such as cancer^9,10^. Therefore, it is crucial to study the SVs at single-cell resolution. Based on the third-generation sequencing (TGS, also known as single-molecule sequencing) platforms, we developed single-molecule real-time sequencing of long fragments amplified through transposon insertion (SMOOTH-seq)^11^ in recent years, which used low-density Tn5 transposase to randomly fragment genomic DNA from an individual cell to achieve relatively even genome amplification. It could efficiently detect the structural variations in addition to CNVs and SNVs. However, limited coverage of both alleles in a diploid cell made it of high false negative rates in the detection of heterozygous single-nucleotide polymorphisms (hetSNPs).

**Fig. 1:**
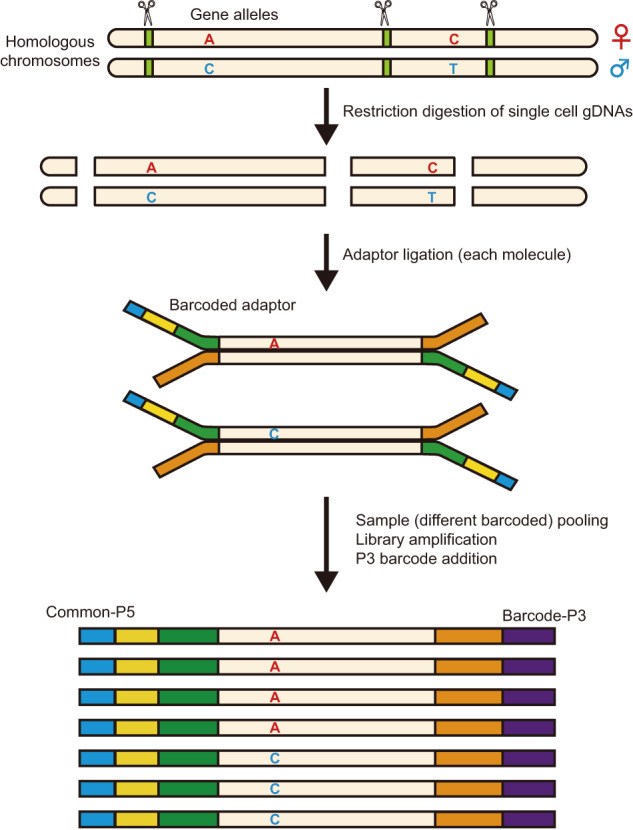
The schematic of Refresh-seq. After single cell lysis and proteinase digestion, restriction digestion of single cell genomic DNAs (gDNAs), end repair and dA-tailing afterwards are performed on a small volume. Then dsDNA adapters are ligated to 3’-dA-tailed molecules. Barcoded adaptors are used in Refresh-seq (multiplexed) and cells with different barcodes are pooled together and then purified. The purified samples are amplified to generate enough DNA material for Nanopore sequencing with another barcode (P3 barcode) addition.

In SMOOTH-seq, two alleles (for example, A and B) in homologous chromosomes are cut randomly. If the genome coverage is n% (n% chance of capturing the allele A or allele B), the possibility of capturing both allele A and B simultaneously should be n% × n%, i.e., (n%)^2^. Note that the two alleles in the diploid genome usually have identical restriction endonuclease recognition sites; the homologous DNA fragments generated by restriction endonuclease cutting usually have identical length (Fig. 1). Therefore, in DNA fragments generated by restriction endonuclease cutting, the possibility of capturing both alleles would be higher because fragments with equal length have more chance to be amplified simultaneously than those with random lengths. Based on this assumption, we developed Refresh-seq (restriction fragment ligation-based genome amplification and TGS), a novel single-cell long-read whole-genome sequencing technique based on restriction endonuclease cutting and ligation strategy (Fig. 1 and Supplementary Fig. S1a). Restriction endonucleases were identified in the early 1950s, subsequently being widely used in molecular biology of DNA such as physical DNA mapping, DNA manipulation and DNA accessibility studies^12^. For example, *Msp* I was used in multiplexed single-cell reduced representation bisulfite sequencing (Msc-RRBS) to enrich CpG-rich sequences such as CpG islands^13^. Restriction enzymes were also used to reduce the complexity across target genomes in restriction-site associated DNA sequencing (RADseq) to deliver high-resolution population genomic data^14^, whereas no trial has been made to combine restriction endonucleases with TGS platform-based whole-genome sequencing. We developed Refresh-seq and investigated the great potential of restriction endonucleases in whole-genome amplification for the first time.

Meiosis is a crucial process in generating haploid gametes for sexual reproduction and serves as the basis of genetic diversity^15^. Crossover represents the exchange between homologous chromosomes and promotes a new combination of haplotypes from parents on recombinant chromosomes in the offspring^15^. Linkage disequilibrium and pedigree studies have revealed that in all the studied organisms the distribution of crossovers is uneven across the genome, but recombination active regions are not conserved between species^16,17^. Also, recombination rates differ between the two sexes in many organisms^18,19^, showing sexual dimorphism. Previous studies based on genetic linkage analyses can only analyze the crossover patterns of the gametes that can generate live offspring. Currently it lacks single-cell whole-genome sequencing analyses revealing and comparing the sexual dimorphism in meiotic recombination of the same mouse strain simultaneously. Here, we applied Refresh-seq to map meiotic crossovers in both male and female mouse gametes with high resolution at low sequencing depth accompanied by accurate detection of aneuploidy. More importantly, we took the advantage of TGS long reads to phase the heterozygous SVs (hetSVs) with high precision. Our results show that Refresh-seq has great potential for biological and medical applications.

## Results

### The schematic of Refresh-seq

To investigate the feasibility of Refresh-seq, we first tested it on 500 pg, 100 pg, 50 pg and 10 pg of genomic DNAs extracted from GM12878 (HG001) cells, a human diploid B lymphoid cell line.

Firstly, we simulated the length distribution of DNA fragments after restriction endonuclease cutting and validated the accuracy by applying to bulk genomic DNAs and downstream fragment analysis. Then we ligated adaptors and amplified the DNA fragments and found that the long DNA fragments over 3 kb had lower recovery rate probably due to the efficiency of ligation reaction. Thus, we should choose endonuclease that generates DNA fragment length concentrated between 1 kb and 3 kb to get better whole-genome coverage of single cells. Also, due to the amplification bias of shorter DNA fragments, the fragments with concentrated length distribution would be amplified more evenly and the endonuclease could consequently perform better for single-cell whole-genome sequencing. Therefore, *Eco*RI was chosen due to the relatively concentrated and proper (most fragments between 1 kb and 3 kb) length distribution of genomic fragments after restriction fragment length simulation (Supplementary Fig. S1b–d). Refresh-seq worked best on 500 pg of genomic DNAs and achieved ~50% genome coverage with ~1× sequencing depth. The samples started with 10 pg of genomic DNAs achieved ~9% genome coverage with ~1× sequencing depth. An individual human cell contains about 6 pg of genomic DNAs and we then tested Refresh-seq on the scale of genomic DNAs of a single cell. But it didn’t work well until the volume of digestion and amplification system was reduced to 1/10 of the original trial, which increased the relative concentration of genomic DNAs in the reaction. Briefly, restriction digestion of single-cell genomic DNAs and afterward end repair and dA-tailing were performed in small volume after single-cell lysis. Then dsDNA adapters were ligated to 3’-dA-tailed molecules to provide complementary DNA sequence of barcode addition (Supplementary Fig. S1a). Over amplification was observed when the numbers of PCR cycles exceeded 20. Thus the second round of amplification could be applied to the PCR products after purification. Multiplexed version of this method, referred to as Refresh-seq (multiplexed), was also developed, where barcoded adaptors were used to increase experimental throughput accordingly. After end repair and dA-tailing, single-cell genomic DNA fragments were ligated with barcoded adaptors. Cells with different barcodes were then pooled together, after which library amplification with P3-barcode addition was performed to generate enough DNA for nanopore sequencing (Fig. 1).

We performed species mixing experiments for Refresh-seq (multiplexed), using two cell lines mixed in equal quantities, including a human cell line (GM12878) and a mouse cell line (3T3). In Refresh-seq (multiplexed), none of the single cells were identified as doublets after quality control (Supplementary Fig. S1e), which meant that minimal cross contaminations were observed in Refresh-seq (multiplexed).

### Refresh-seq had increased genome coverage and uniformity at single-cell level

We comprehensively compared Refresh-seq with SMOOTH-seq, the first single-cell genome sequencing method based on TGS platform^11^. Refresh-seq was performed on 173 human NA24385 (HG002) cells, with 88 cells using the single tube version (Refresh-seq) and 85 cells using the multiplexed version (Refresh-seq (multiplexed)). Single-cell genome sequencing data of HG002 by SMOOTH-seq were from our previous study^20^.

**Fig. 2:**
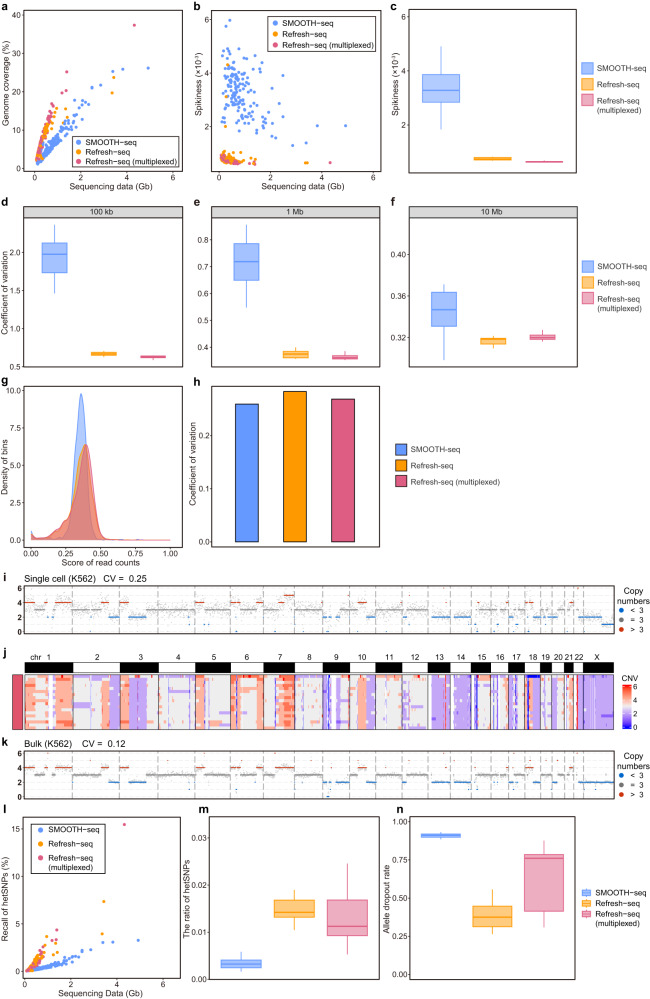
Comparison of Refresh-seq with SMOOTH-seq. **a** Scatter plot showing the sequencing data and genome coverage of each HG002 cell. Cells amplified with SMOOTH-seq were shown as blue dots. Cells amplified with Refresh-seq (single tube version) were shown as orange dots. Cells amplified with Refresh-seq (multiplexed) were shown as red dots. **b** Scatter plot showing the sequencing data and Spikiness value of each HG002 cell. **c** Boxplot showing the quantification of Spikiness values at ~0.25× sequencing depth shown in **b**. For boxplots, center line represents the median; box limits represent upper and lower quartiles. **d**–**f** CVs for read depths along the genome under 100 kb, 1 Mb and 10 Mb bins, respectively, showing amplification bias on different scales. **g** The density of bins with normalized read depths in pseudobulk (~13×) from SMOOTH-seq, Refresh-seq and Refresh-seq (multiplexed). **h** CVs of the read depths in pseudobulk (~13×) from SMOOTH-seq, Refresh-seq and Refresh-seq (multiplexed). **i** CNVs of one single K562 cell showing in 1 Mb windows. **j** CNVs for 18 single K562 cells amplified with Refresh-seq showing on heatmap. **k** CNVs of bulk K562 samples showing in 1 Mb windows. **l** Scatter plot showing the sequencing data and the detection of both alleles at hetSNP sites of each cell. **m** Quantification of the ratios of hetSNPs detected by SMOOTH-seq, Refresh-seq and Refresh-seq (multiplexed). **n** Allele dropout rates of SMOOTH-seq, Refresh-seq and Refresh-seq (multiplexed) at hetSNP sites covered by over 5 reads.

Refresh-seq had 69.7% of reads aligned to the human genome. The average number of reads for each single cell was 221,203, and the average length of reads was 1669 bp. Refresh-seq (multiplexed) had a mapping ratio of 88.4%. The average number of reads for each single cell was 188,639 with an average length of 2172 bp. Refresh-seq could achieve relative high genome coverage at low sequencing depth and we compared the coverage of Refresh-seq with that of SMOOTH-seq. Refresh-seq had better coverage than SMOOTH-seq at the same sequencing depth and it required fewer sequencing data to reach the same genome coverage (Fig. 2a). With ~0.25× sequencing depth, Refresh-seq achieved ~11.7% genome coverage and Refresh-seq (multiplexed) achieved ~13.3% genome coverage, compared with 6.0% in SMOOTH-seq. The coverage of Refresh-seq and Refresh-seq (multiplexed) increased linearly with the increase of sequencing data at low sequencing depth (Supplementary Fig. S2a). The coverage of Refresh-seq (multiplexed) outperformed Refresh-seq, probably due to the higher initial genomic input (carrier effect) which led to less amplification bias (Fig. 2a and Supplementary Fig. S2a).

Now that single-cell whole-genome sequencing methods enable us to trace the amount of genomic DNA from a single cell (a few picograms), amplification uniformity is a quite important feature and is crucial for accurate measurements of genomic variations such as CNVs. Although SMOOTH-seq utilized Tn5 transposase, which was considered to relatively randomly fragment genomic DNAs from single-cell lysates, raw reads of Refresh-seq distributed more evenly compared with SMOOTH-seq at chromosome level (Supplementary Fig. S2b). It meant that Refresh-seq, based on specific restriction endonuclease recognition site, actually had better amplification uniformity than SMOOTH-seq. We then compared the single-cell amplification uniformity between these two methods in three ways.

The first approach was to calculate the bin-to-bin variations in read counts (Spikiness), the Bhattacharya distance between the number of continuous stretches of bins with the same copy number^21^. Lower Spikiness scores meant fewer variations and better uniformity. Both Refresh-seq and Refresh-seq (multiplexed) had better Spikiness scores than SMOOTH-seq (Fig. 2b, c). Three cells amplified with Refresh-seq showed comparable uniformity with SMOOTH-seq and they featured low concentrations after amplification. Spikiness scores of SMOOTH-seq were 4.3 times higher than Refresh-seq and 5.2 times than that of Refresh-seq (multiplexed) at ~0.25× sequencing depth, which validated that Refresh-seq had better amplification uniformity (Fig. 2b, c). The second approach used coefficient of variation (CV) of read density, which was also a nice measurement of the uniformity of the read distribution. We calculated CVs for each single cell and Refresh-seq showed less variations as well (Supplementary Fig. S2c). CVs of SMOOTH-seq were 1.85 times higher than Refresh-seq and 1.90 times higher than those of Refresh-seq (multiplexed) at ~0.25× sequencing depth (Supplementary Fig. S2d). The third approach was to calculate the Shannon entropy for read counts and Refresh-seq also had better performance than SMOOTH-seq (Supplementary Fig. S2e, f), consistent with the results of Spikiness and CVs.

In order to measure the uniformity at different scales, we further calculated CVs of read density at ~0.25× sequencing depth under different sizes of bins. The genome was divided into 100 kb, 1 Mb and 10 Mb bins respectively and the number of reads in each bin was calculated. The CVs of read number were used to quantify the amplification bias at different scales. As shown in Fig. 2d–f, Refresh-seq achieved lower CVs than SMOOTH-seq with respect to all bin sizes, which proved that Refresh-seq had better amplification uniformity.

We validated that Refresh-seq had better amplification uniformity than SMOOTH-seq at the single-cell level. Then we merged single-cells into pseudobulk and calculated the uniformity at the 1 Mb scale as described above (Fig. 2g, h). Pseudobulk samples were adjusted to ~13× mean sequencing depth for further comparison. The density of bins in SMOOTH-seq had sharper peaks than Refresh-seq and Refresh-seq (multiplexed) (Fig. 2g), indicating that the number of reads in different bins of SMOOTH-seq had fewer variations. SMOOTH-seq also featured slightly lower CV values of read counts at pseudobulk level (Fig. 2h). It was reasonable due to the coincident distribution of restriction endonuclease recognition sites in each cell. In Refresh-seq, restriction fragments with appropriate length distribution (within 3 kb) tended to be amplified in each single cell. When reads from different cells were merged as pseudobulk, these regions were enriched and exhibited accumulation of reads. Pseudobulk sample of Refresh-seq achieved ~83.5% genome coverage while SMOOTH-seq could cover 93.6% of the genome, which was also a reflection of read enrichment.

Amplification uniformity is important for measurements of CNVs. We then performed Refresh-seq on 18 human K562 cells to analyze the CNVs at low sequencing depth (~0.4×). We calculated the ratios of reads in every window at 1 Mb size within each individual cell. The CNV patterns were relatively stable for single cells and the coefficient of variation was 0.24 on average (Fig. 2i). The patterns were clearly visible in the heatmap (Fig. 2j) and were consistent with those in bulk samples (Fig. 2k). Thus, Refresh-seq had acceptable performance on CNV analysis at the resolution of 1 Mb.

### Refresh-seq had lower allele dropout rate than SMOOTH-seq

Allele dropout arises from the tiny amounts of input material from a single cell and uneven amplification, which largely restricts the application of single-cell whole-genome sequencing methods particularly for medical applications^1^. We compared the recall of hetSNPs in Refresh-seq and SMOOTH-seq to reflect the ability to detect both alleles in a diploid cell. Cells amplified with Refresh-seq and Refresh-seq (multiplexed) had higher recall of hetSNPs compared with SMOOTH-seq (Fig. 2l, m). With ~0.25× sequencing depth, Refresh-seq detected ~1.64% of hetSNPs, which was 5 times higher than ~0.33% of hetSNPs by SMOOTH-seq (Fig. 2m). In hetSNP positions covered by more than 5 reads, the mean allele dropout rate of Refresh-seq was 38% and that of Refresh-seq (multiplexed) was 65%, which were significantly lower than 90% in SMOOTH-seq (Fig. 2n). It could be that the two alleles on the homologous chromosomes were cut into DNA fragments with identical length which tended to be amplified simultaneously. Refresh-seq (multiplexed) featured higher allele dropout rate than the single tube version (Fig. 2n), which might be due to the lower ligation efficiency of the self-synthesized barcoded adaptors.

To test the universality of Refresh-seq and Refresh-seq (multiplexed), Refresh-seq was performed on 37 human HG001 cells and Refresh-seq (multiplexed) was performed on 120 HG001 cells. Refresh-seq and Refresh-seq (multiplexed) had consistent performance on HG001 cells compared with HG002 cells. With ~1× sequencing depth, Refresh-seq for HG001 achieved ~21.4% genome coverage and Refresh-seq (multiplexed) achieved ~24.6% genome coverage (Supplementary Fig. S2g). With ~1× sequencing depth, Refresh-seq detected ~6.6% of hetSNPs (Supplementary Fig. S2h). The allele dropout rate of Refresh-seq was 48%, and that of Refresh-seq (multiplexed) was 64% in hetSNP positions covered over 5 times. Refresh-seq using two other restriction endonucleases, *Sac*I and *Asi*SI, was performed on both HG001 (Supplementary Fig. S2g, h) and HG002 (Supplementary Fig. S2i, j) cells. The predicted fragment length distribution of *Sac*I cutting was similar to that of *Eco*RI (Supplementary Fig. S1c). The average length was 1738 bp/1837 bp in Refresh-seq (*Sac*I) and 2058 bp/2829 bp in Refresh-seq (multiplexed) (*Sac*I) for HG001/HG002 cells, respectively. Refresh-seq using *Eco*RI and *Sac*I had comparable genome coverage and recall of hetSNPs, illustrating the reproducibility and universality of Refresh-seq technique (Supplementary Fig. S2g–j). *Asi*SI had longer (8 bp) recognition sequence which sparsely distributed throughout the genome and the predicted length of DNA fragments was much longer (Supplementary Fig. S1d). Now that only fragments with appropriate length range (usually between 1 kb and 3 kb) could be successfully amplified, these genomic regions would be enriched by Refresh-seq. Given that only part of genome could be retained as input, only Refresh-seq (multiplexed) might work well for the restriction endonucleases with longer recognition sequences. The coverage of Refresh-seq (multiplexed) (*Asi*SI) was low due to the enrichment of DNA fragments of specific lengths (Supplementary Fig. S2g, i), but it acquired much higher sequencing depth in these recovered genomic regions (Supplementary Fig. S2k), which was similar to RADseq^22^. Therefore, Refresh-seq using endonucleases of long recognition sequences could achieve higher sequencing depth when sequencing the same amount of data with the selection of the length of DNA fragments.

### Refresh-seq of mouse sperm to phase the haploid genome

**Fig. 3:**
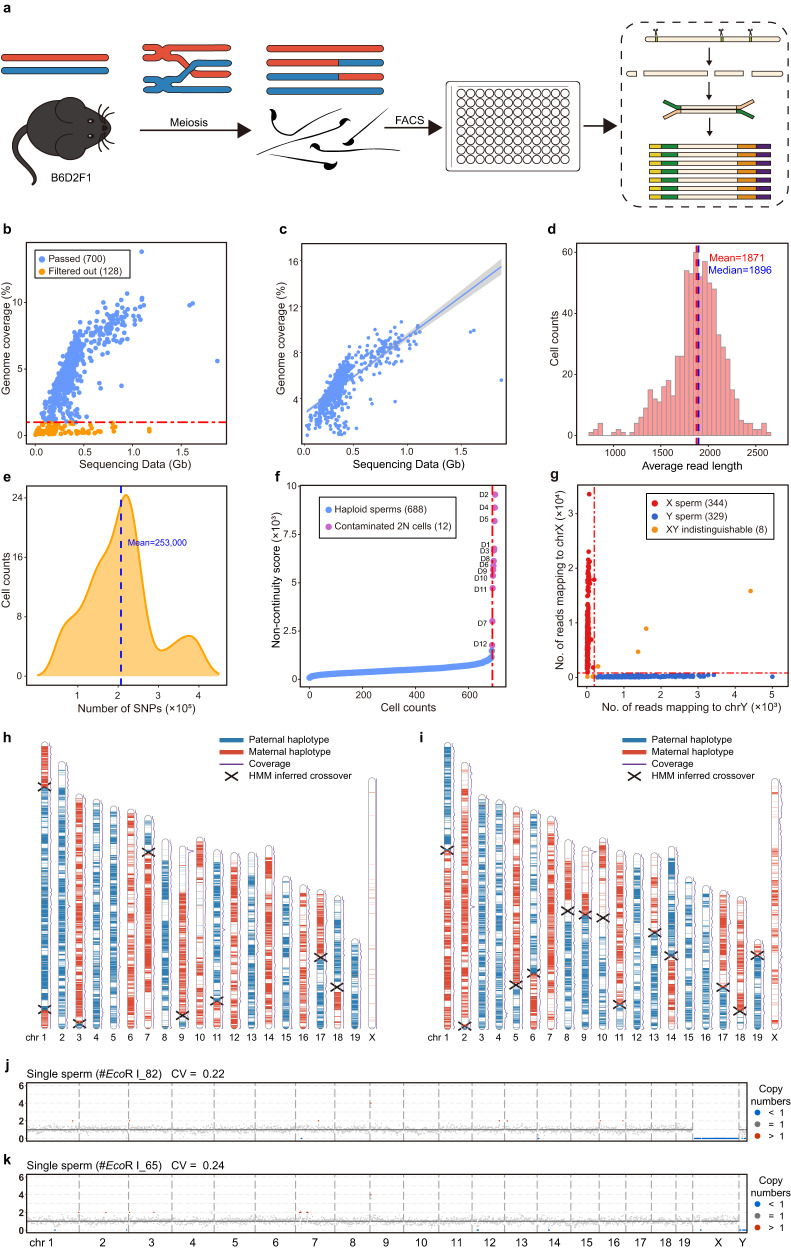
Schematic charts of mouse sperm meiosis and phasing of genome using single-sperm Refresh-seq data. **a** Schematic charts showing the process of meiosis of hybrid mouse sperm and Refresh-seq of single sperm. Mature sperm cells of B6D2F1 (B6 × DBA F1 hybrid) mice undergone meiotic homologous recombination were obtained and sorted to each well by fluorescence-activated cell sorting (FACS). Refresh-seq was performed to each single sperm. **b** Scatter plot showing the sequencing data and genome coverage of each sperm cell. Cut off was set at genome coverage of 1% marked as red dotted line. **c** Scatter plot showing the sequencing data and genome coverage of each sperm cell passed quality control fitted with linear regression (line) at 95% credible interval (shading). **d** Distribution of average read-length for single sperm amplified by Refresh-seq. The mean and median lengths are marked as red and blue dotted lines respectively. **e** Distribution of the number of hetSNPs covered in each sperm. The mean number of the covered hetSNPs is marked as blue dotted line. **f** Computational recognition of diploid cells by the non-continuity scores of each sperm (exclude the autosome with the highest frequency). Non-continuity scores of contaminated diploid cells are much higher than haploid sperm. The dashed red line marks the inflection point beyond which sperm cells are flagged as potential diploid cells showing as purple dots and excluded from downstream analysis. **g** Distinction of X sperm cells and Y sperm cells using number of reads mapping to X and Y chromosomes. 344 X sperm cells (red dots) and 329 Y sperm cells are identified, with 8 sperm cells being indistinguishable for X or Y (orange dots). **h**, **i** Parental haplotype contribution map of the 20 chromosomes from individual sperm cells. Parental haplotype contributions are determined by the proportion of the paternal or maternal SNPs, and crossover positions are detected by identifying the crossing locations of the two parental haplotypes by an HMM. Parental haplotype contribution map of the 20 chromosomes from one Y sperm cell is shown in **h** and map of one X sperm cell is shown in **i**. Blue regions are contributed by the paternal SNPs and red regions are contributed by the maternal SNPs. Crossover sites are marked as forks. **j** CNVs of the Y sperm displayed in **h**, showing in 1 Mb windows. **k** CNVs of the X sperm displayed in **i**, showing in 1 Mb windows.

Meiotic recombination is the basis of genetic diversity and is essential for accurate segregation of homologous chromosomes^15^. It results in the exchange of genetic information and thus each germ cell has unique genome sequences. Sequencing multiple single germ cells from an individual allows us to construct the recombination map and have a better understanding of recombination events^23^. We performed Refresh-seq (*Eco*RI) on 828 sperm cells from the male B6D2F1 (C57BL/6NCrl × DBA/2NCrl F1 hybrid) mice which contained 4.3 million hetSNPs to phase the genome and construct the recombination map at high resolution (Fig. 3a). In total, 676 sperm cells were amplified with Refresh-seq (single tube version) and 152 sperm cells were amplified with Refresh-seq (multiplexed). Refresh-seq and Refresh-seq (multiplexed) did not exhibit differences in the detection of the crossover events, and thus downstream analysis was performed without distinguishing different versions of Refresh-seq. With the 828 spermatozoa sequenced at ~0.1–0.3× depth, 700 sperm cells passed quality control with genome coverage higher than 1% (Fig. 3b). The genome coverage increased near-linearly with the increase of sequencing data ranging from 0.1–1 Gb and the mean coverage was about 5% (Fig. 3c). The average length of sequencing reads was 1.9 kb (Fig. 3d) and the average number of reads per sperm was 143,914.

Haplotype means the combinations of genetic variants (or genetic polymorphisms) along a single chromosome that are inherited together from a single parent^24^. Haplotype phasing is crucial for the correct description of the genome and is essential for identification of meiotic crossovers. We phased the genome of each sperm by the hetSNPs of the male mice with high accuracy. The benchmark set of the B6D2F1 hybrid mouse contained 4.3 million hetSNPs from 19 autosomes and X chromosome with relative even distribution (Supplementary Fig. S3a). In each sperm, an average of ~253,000 hetSNPs were detected (Fig. 3e) and the accuracy of SNP detection was over 98.9%. Hidden Markov Model (HMM) was used to phase the hetSNPs and 99.97% of the hetSNPs were precisely phased to the correct haplotype in the genome (Supplementary Table S1), which was comparable with previous studies. The SNP linkage in the gametes offers information for constructing chromosome-level phased haplotypes of the individual^25^. Then, we inferred crossovers on each chromosome of every single sperm with the phased haplotypes. Crossover events in each sperm were identified as transitions between one parental genotype to the other.

We then defined the ‘non-continuity score’. We divided the genome into several genotype regions with corresponding hetSNPs: if a genomic region had consecutive maternal hetSNPs it would be judged as a genomic region of maternal genotype and vice versa. The ‘non-continuity score’ was defined as the frequency of switching between one genomic region of a parental genotype to that of the other parental genotype. The score was generally small in a haploid cell as it just rose at the location of crossover or PCR/sequencing errors and was much higher in a diploid cell with a mixture of two distinct haploid genomes. Due to the existence of two alleles, the non-continuity score of a diploid cell could be aberrantly high when allele dropout happened. Thus, we identified the diploid cells by calculating the non-continuity scores, excluding the autosomes with the highest scores (given the possibility of gain of one chromosome) in each cell^26^ (Fig. 3f). Among the 700 sperm cells passed quality control, there were 688 haploid spermatozoa cells and 12 diploid cells, which could be contaminated diploid somatic cells (Fig. 3f). We labeled the diploid cells as D1 to D12 (Fig. 3f) and validated the authenticity of the 12 diploid cells using distribution maps. The distribution map and CNV patterns of the diploid cell D8 were shown in Supplementary Fig. S3b, c. The map featured balanced distribution of paternal and maternal alleles, illustrating that the cell D8 was likely to be a diploid somatic cell of the B6D2F1 hybrid mouse. We then distinguished X sperm cells and Y sperm cells according to the number and ratio of reads mapping to the X and Y chromosomes (Fig. 3g and Supplementary Fig. S3d). We identified 344 X sperm cells and 329 Y sperm cells, with 8 sperm cells being indistinguishable (sex chromosome gain or loss) (Fig. 3g and Supplementary Fig. S3d). The ratio of X sperm and Y sperm was close to 1:1, which was consistent with Mendel’s Law of Segregation^27^.

We constructed crossover distribution maps of 20 chromosomes for all the sperm cells and showed the crossover maps and CNVs of one Y sperm cell and one X sperm cell (Fig. 3h–k). Overall, Refresh-seq could acquire high-resolution crossover distribution map with relative low sequencing depth.

### Detection of aneuploid sperm cells with high accuracy

**Fig. 4:**
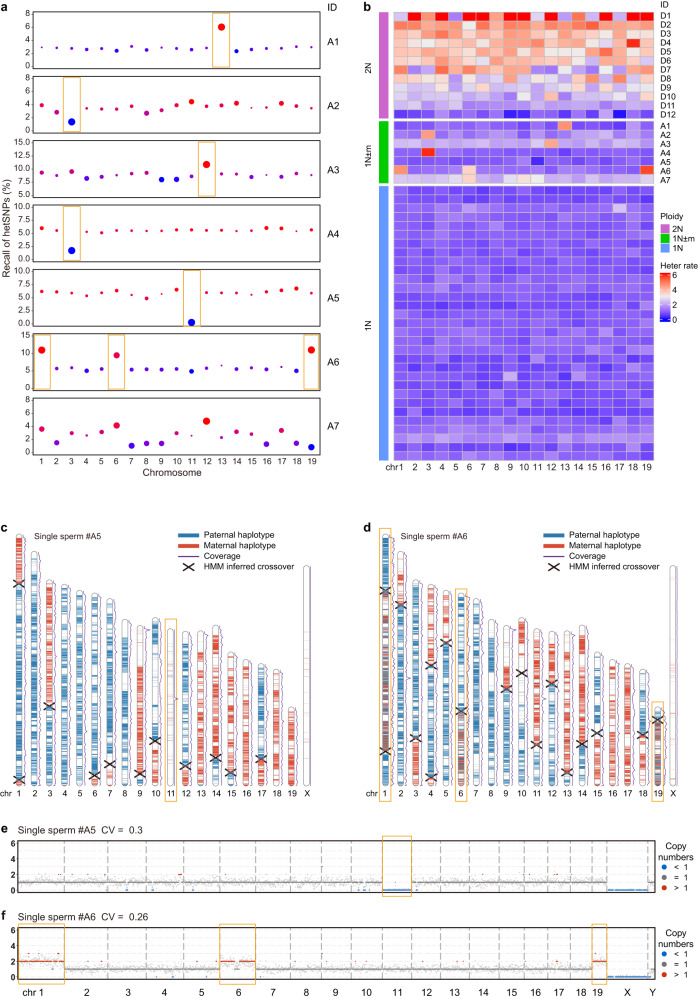
Identification of aneuploid sperm. **a** The ratios of covered hetSNPs compared with the gold standard in 19 autosomes from 7 aneuploid sperm cells. Outlier blue dots indicate loss and red dots indicate gain of chromatids. The sizes of the dots mean the deviation from the average ratio. The verified aneuploid chromosomes are highlighted by orange rectangles and the sperm A7 is more likely to be an unevenly amplified sample (technical artefact) rather than true aneuploidy. **b** The rates of covering both alleles at hetSNP sites in sperm cells. Heatmap showing the heterozygosity rates of the 19 autosomes from 12 diploid cells (showing as 2 N), 7 aneuploid sperm cells (1 N ± m) and several haploid sperm cells (1 N). The sperm A7 is more likely to be an unevenly amplified sample (technical artefact) rather than true aneuploidy. **c** Parental haplotype contribution map of the 20 chromosomes from the aneuploid sperm cell A5 with loss of chromosome 11. Blue regions are the paternal SNPs and red regions are the maternal SNPs. Crossover sites are marked as forks. **d** Parental haplotype contribution map of the 20 chromosomes from the aneuploid sperm cell A6 with gain of chromosomes 1, 6 and 19. Blue regions are the paternal SNPs and red regions are the maternal SNPs. Crossover sites are marked as forks. **e** CNVs of the aneuploid sperm A5, showing in 1 Mb windows. **f** CNVs of the aneuploid sperm A6, showing in 1 Mb windows.

Aneuploidy is usually due to errors of chromosome segregation during cell division, leading to abnormal number of chromosomes in a cell. Aneuploidy in early embryos often leads to miscarriage or genetic disorders of fetus. Several screening and diagnostic technologies such as CGH and SNP arrays have been developed to detect aneuploidy but the resolution is relatively limited^28^. The aneuploidy could be determined using MALBAC due to its high genome coverage and uniformity^29^. Using Refresh-seq, autosome aneuploidy could be accurately screened and verified with three approaches due to its high coverage uniformity and low allele dropout rate. First, aneuploid sperm cells with gain of chromatids were screened out with the non-continuity scores in different chromosomes (Supplementary Fig. S4a–h). Diploid cells showed high non-continuity scores in most chromosomes (Supplementary Fig. S4a–h), whereas aneuploid sperm cells featured elevation of non-continuity scores in aneuploid chromosomes (Supplementary Fig. S4c–h). We identified five sperm cells (labeled as A1, A2, A3, A4, A6) with gain of chromatids by calculating the non-continuity score of each chromosome, with A6 having gain of three chromatids (chromosomes 1, 6 and 19) (Supplementary Fig. S4c, e, h). Secondly, loss of chromatids was often characterized by a significant reduction in the recall of hetSNPs on the aneuploid chromosomes compared with the relative homogeneous proportion of hetSNPs in other normal chromosomes (Fig. 4a). We identified 4 sperm cells with chromatid loss (labeled as A2, A4, A5, A7) (Fig. 4a), among which A2 and A4 also showed gain of another chromatids (both on chromosome 3) by calculating the non-continuity scores. The sperm A7 did not show homogeneity of hetSNP recall in most chromosomes, which was not likely to happen in a cell, indicating that the sperm A7 was more likely to be an unevenly amplified sample (technical artefact) rather than being an aneuploid cell. The sperm cells with gain of chromatids (A1, A3 and A6) found before by non-continuity scores also showed higher detection of heterozygous SNPs in the corresponding aneuploid chromosomes (Fig. 4a). Thirdly, since Refresh-seq had comparative efficiencies in detecting both alleles in a cell, aneuploid sperm cells with gain of chromatids were more likely to have both alleles detected, showing increased heterozygosity rates of the gained chromosomes. In the screened sperm cells with gain or loss of chromatids, the heterozygosity rates of the aneuploid chromosomes were clearly distinguishable from other normal chromosomes except for A7 which was likely to be unevenly amplified (technical artefact) (Fig. 4b). The diploid cells (except for D11 and D12 which had the lowest coverages in diploid cells) had higher heterozygosity rates in most chromosomes while aneuploidy featured changed heterozygosity rates of the aneuploid chromosomes (Fig. 4b). The aneuploid chromosomes identified by heterozygosity rates were the same with those identified by non-continuity scores (Supplementary Fig. S4a–h) and the recall of hetSNPs (Fig. 4a) as expected. For example, the heterozygosity rate, recall of hetSNPs and non-continuity score of chromosome 13 all elevated in the sperm A1, illustrating gain of chromosome 13 in this sperm. We further confirmed the authenticity of the 6 aneuploid sperm cells (A1–A6) using crossover distribution maps and CNV patterns, whereas A7 was an unevenly amplified sample (technical artefact) as expected. For the sperm A5 shown in Fig. 4c, loss of chromosome 11 was clearly observed while the other chromosomes showed normal coverage depth. The CNV patterns of this sperm showed that the copy number on chromosome 11 was less than 1 (Fig. 4e), consistent with the crossover distribution map as well as recall of hetSNPs and heterozygosity rates. Another sperm A6 whose crossover distribution map shown in Fig. 4d featured gain of chromosomes 1, 6 and 19, and the SNP map showed higher heterozygosity on these chromosomes. The CNV patterns of the sperm showed gain of copy number on chromosomes 1, 6 and 19 as well (Fig. 4f). For aneuploid sperm cells A2 and A4, they both had gain at the top quarter of chromosome 3 and loss at other regions of the chromosome, which could be clearly observed in the haplotype contribution map (Supplementary Fig. S4i, j). In the CNVs of chromosome 3, we could see copy numbers higher than 1 (red) in regions having gain of chromosomes and the copy numbers came to zero where loss of chromosomes happened (Supplementary Fig. S4k, l). The co-occurrence of gain and loss on the same chromosome also explained for the different changes in recall of hetSNPs and heterozygosity rates (loss inferred by recall of hetSNPs and gain inferred by heterozygosity rates).

In conclusion, we identified 8 sperm cells with sex chromosome gain or loss and 6 sperm cells with autosome aneuploidy. The ratio of aneuploid sperm cells was 2% in our data and was consistent with previous studies (1%–2%)^30^. Thus, Refresh-seq could distinguish chromosome-level aneuploidy as well as gain or loss of chromosome fragments with high accuracy and sensitivity.

### Genetic map of mouse sperm and crossover interference

**Fig. 5:**
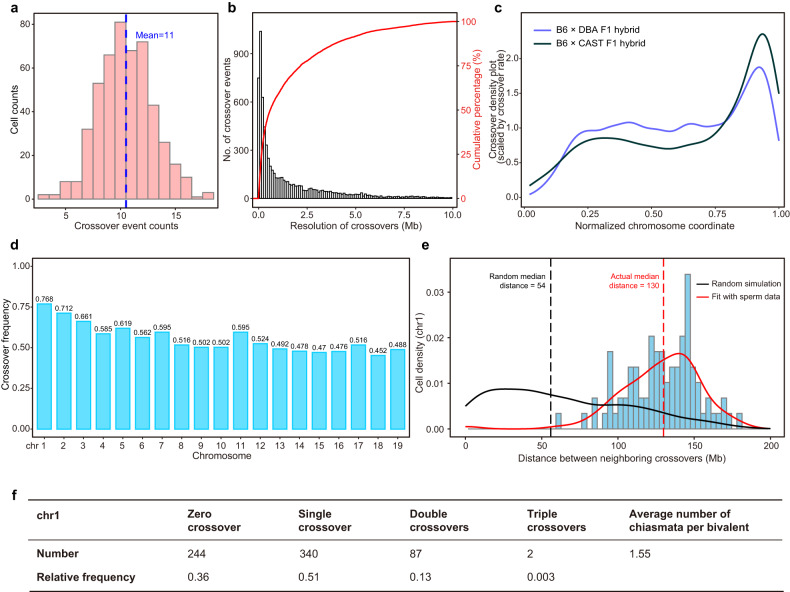
Distribution characteristics of crossovers in mouse sperm. **a** Distribution of the number of autosomal crossover events identified per sperm. The mean number of crossovers is marked as the blue dotted line. **b** Resolution of crossover determination at sequencing depth of ~0.1–0.3×. Accumulative percentage of crossover numbers is shown as red curve. **c** Crossover location density plots for all chromosomes. The *x* axis shows the normalized chromosome coordinate in which ‘0’ means the top of chromosomes and ‘1’ means the bottom of chromosomes. It shows the crossover density by the distances from the centromere to telomere. The blue curve shows the distribution of crossovers in our data of B6 × DBA F1 hybrid sperm and the black curve shows the distribution in data of C57BL/6J × CAST/EiJ F1 hybrid sperm from the previous study^31^. **d** Average number of crossovers identified per chromosome per sperm, showing ~0.5 crossovers per haploid sperm (indicating at least one crossover per chromosome pair per meiosis). **e** Crossover interference on chromosome 1 of male meiosis. Distance between two crossover events on the same chromosome 1 is measured in megabases of DNA length. The red line represents the distribution curve fitting the experimental data. The black line shows the distribution of randomly generated distances. The dotted lines represent the median distance of the random data and experimental data. **f** Distribution of crossover classes along chromosome 1.

Crossover events in each sperm were identified as transition between the parental haplotypes as described above. We identified a total of 7292 crossovers in 673 euploid sperm cells, with an average of 11 recombination events in each sperm (Fig. 5a), which was compatible with previous study^31^. Crossover locations were inferred with a median resolution of ~400 kb (Fig. 5b). Approximately 50%, 40% and 25% of the crossovers could be assigned to intervals of 300 kb, 200 kb, and 100 kb, respectively. Thus, Refresh-seq could achieve relatively high resolution in crossover identification with low sequencing depth (~0.1–0.3×). All sperm cells tended to concentrate crossovers in specific regions of the genome and the enrichments of crossover locations were generally consistent with previous data of B6 × CAST F1 hybrid^31^, with more crossovers in distal regions (enriched near the telomeres) and fewer in centromere-proximal regions (Fig. 5c), agreeing with previous studies^32^. An explanation for this could be crossovers near centromeres might interrupt the attachment of the spindles to the centromeres or disrupt the pulling of the centromeres to opposite poles of a cell during cell division^33^. Crossover location density plots were described for each chromosome (Supplementary Fig. S5a), showing the specific spatial enrichments of crossovers in different chromosomes. It had been reported that DNA double-strand break (DSB) formation occurred after DNA replication in the process of meiosis and recombination was highly related to DNA replication at the scales of time and space^34^. In our data, the positions with high crossover density corresponded with genomic regions of early DNA replication and positions with low crossover density corresponded with genomic regions of late DNA replication (Supplementary Fig. S5b).

We calculated the crossover frequency of each chromosome and chromosome 1 featured the highest frequency due to its longest chromosome axis (Fig. 5d), consistent with previous studies^31^. At least one crossover per chromosome pair per meiosis was available to ensure the correct separation of homologous chromosomes, equivalent to 0.5 crossovers per chromosome pair per sperm (Fig. 5d)^35^. In our sperm data, a nearly linear correlation between chromosome size and chromosome recombination frequency was observed, with a Pearson correlation coefficient of 0.86 (Supplementary Fig. S5c). The correlation was also validated in previous studies which observed crossovers (MLH1 foci) cytologically^36–38^.

Crossover interference is a phenomenon in which the crossover at one position would reduce the probability of another crossover occurring nearby on the same chromosome^23,29^ and the distance the interference signal spreads is called the ‘interference distance’^36^. We calculated the distances between two crossovers on the same chromosome and compared the distribution with random simulation (Fig. 5e). The median distance between two crossover events on chromosome 1 was 130 Mb, much longer than 54 Mb based on random simulation, and only about 1% of the distances between double crossover events were smaller than 60 Mb (Fig. 5e). A maximum of three recombination crossover events was observed to occur simultaneously on chromosome 1, and the frequencies of zero to triple crossovers (Fig. 5f) were consistent with the previous publication^19^. Crossover interference was also evident for all chromosomes and the median interference distance was 92 Mb, compared with 56 Mb based on random simulation (Supplementary Fig. S5d).

### Detection and phasing of SVs (insertions and deletions) in mouse sperm

SVs are important sources of genetic diversities and contribute to many diseases such as tumor and neurodevelopmental disorders^9,39^. Compared with the NGS platforms, the advantage of the long read-length of the TGS platforms improves the ability of detecting SVs. Not only the detected number but also the detection resolution of SVs have been greatly improved^40^. Therefore, we identified insertions and deletions in our sperm data, which accounted for the second most common types of genomic variations^41^. In order to evaluate Refresh-seq’s ability of SV detection, we used the 54,471 heterozygous SVs (insertions and deletions) in the B6D2F1 hybrid mouse as the gold standard set.

**Fig. 6:**
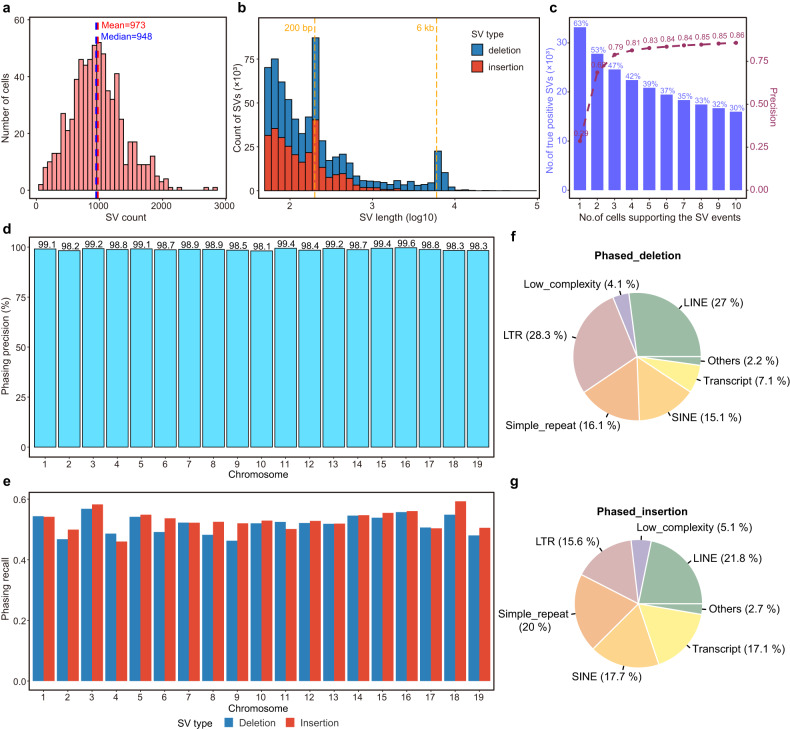
Detection and phasing of SVs in mouse sperm. **a** Distribution of the number of true positive SVs identified per sperm. The mean and median numbers of SVs are marked as red and blue dotted lines respectively. **b** Length distribution of identified true positive SVs. Deletions are shown in blue and insertions are shown in red. Local peaks of SV length are marked as orange dotted lines. **c** Precision of SVs (deletions and insertions) detected by Refresh-seq and the percentage of true positive SVs with different numbers of supporting cells. **d** The genome-wide haplotype phasing precision of SVs on the chromosome scale. **e** The proportion of phased SVs in bulk benchmark SVs set on the chromosome scale. Deletions are shown in blue and insertions are shown in red. **f** The proportion of different types of elements involved in phased deletions. **g** The proportion of different types of elements involved in phased insertions.

In our data of mouse sperm, a total of 33,193 SVs supported by gold standard were detected, including 18,825 deletions and 14,368 insertions. An average of 973 SVs were detected in each sperm (Fig. 6a). The median length of deletions was 180 bp and the median length of insertions was 122 bp (Fig. 6b). A peak at 6–7 kb was visible on the length distribution of deletions, which corresponded to the length of LINE1 elements; and peaks at 200 bp, close to the length of B1 elements (equivalent of Alu elements in human), were clear for both deletions and insertions (Fig. 6b)^42,43^.

We used precision and recall to evaluate Refresh-seq’s ability of SV detection. At the level of over one read per cell, the precision of SVs supported by three cells was 79% and the recall was 47% (Fig. 6c). The precision and recall of deletions were higher than that of insertions and 80.4% of the detected deletions supported by three cells were true (Supplementary Fig. S6a, b). It was reasonable as the average length of Refresh-seq was not long enough to detect large insertions.

SVs had a great diversity between two alleles, leading to loss, gain and reshuffling of genes as well as regulatory elements. If two neighboring SVs in the same gene were on the same allele, the cell would have one normal version of the gene and usually showed no defects. If they happened on different alleles, the cell would lack normal version of the gene and might show disease phenotypes. Haplotype information is important to distinguish between these two scenarios^24^. Thus, haplotype information of SVs can be helpful for studying allele-specific gene expression, heterozygosity, genetic association testing, testing for natural selection, and other diplontic effects^44,45^. In order to phase the SVs accurately, we used SVs supported by over two reads per cell and the accuracy was 76.4%. Of all the phased SVs, 98.8% were precisely phased to the true haplotypes in genome and no significant differences were observed between different chromosomes (Fig. 6d and Supplementary Table S1). The overall recall ratio of true-phased SVs was 50% (Fig. 6e and Supplementary Table S1). We then annotated the types of these correctly phased SVs (Fig. 6f, g) and the overall distribution of elements was similar with SVs in the bulk samples (Supplementary Fig. S6c, d). The proportion of unique sequences in phased SVs was slightly higher than that in bulk gold standard and the proportion of repetitive elements was a bit lower, which might be due to different mapping qualities of unique sequences and repetitive elements in the genome. The counts and recall of the different annotation types of SVs were shown in Supplementary Fig. S6e, f. We showed a paternal (DBA) specific deletion of SINE element between two LINE elements (Supplementary Fig. S6g) and a paternal specific insertion near a simple repeat region (Supplementary Fig. S6h), illustrating the ability of phasing SVs in highly repetitive or low-complexity genomic regions where short-read NGS technologies met problems^46,47^.

### Refresh-seq of mouse polar bodies and probing meiotic recombination of female germ cells

The process of meiosis has significant sexual dimorphism with different characteristics of recombination in males versus females^48^. Meiosis of spermatocytes is a continuous process, whereas oocytes are arrested at the diplotene stage of prophase I after initial meiotic progression. At the time prior to ovulation, oocyte maturation is induced by a surge of luteinizing hormone, after which extrusion of the first polar body (PB1) occurs and the oocyte progresses to metaphase II (MII). The oocyte becomes arrested again at MII until fertilization, when the extrusion of the second PB (PB2) occurs^18,49^. Thus, oocytes are never truly haploid given that the second meiotic division occurs only after fertilization by a sperm cell, which introduces another haploid genome to form the zygote^50^.

**Fig. 7:**
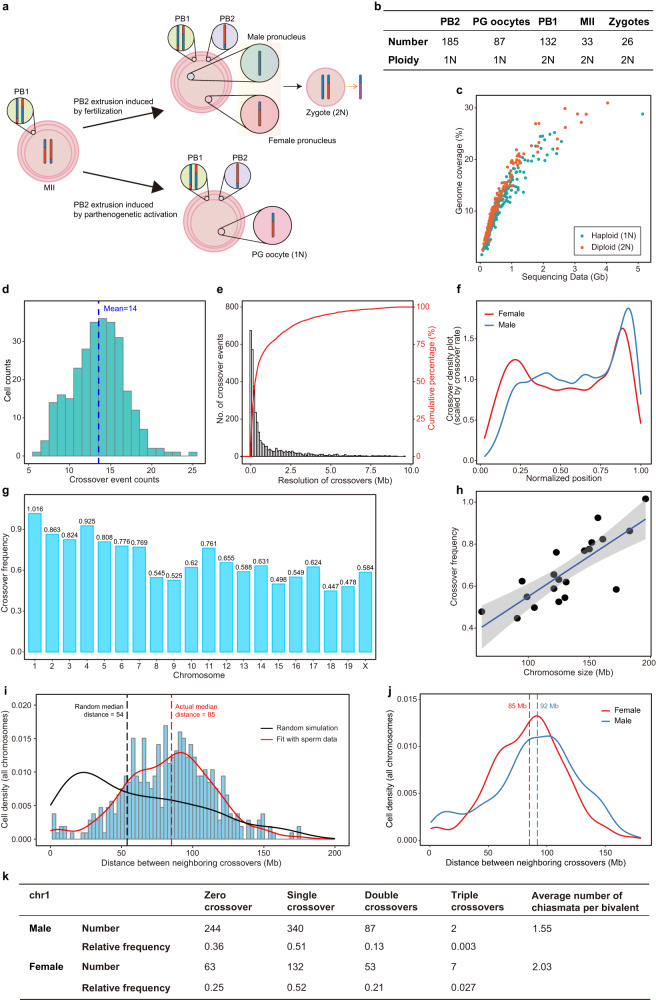
Probing meiotic recombination features of female mice by Refresh-seq. **a** Schematic charts showing the characteristics of meiosis of hybrid female mice. MII oocytes of B6D2F1 which have undergone meiotic homologous recombination are either fertilized with DBA male mice or parthenogenetic activated to induce PB2 extrusion. Haploid PB2, PG oocytes, and diploid PB1, MII, zygotes were obtained and picked up by microcapillary. **b** The numbers of different type of cells and their actual ploidies. **c** Scatter plot showing the sequencing data and genome coverage of each cell. Haploid cells are shown as green dots and diploid cells are shown as red dots. **d** Distribution of crossover numbers in haploid female germ cells including PG oocytes, PB2 and zygotes. The zygotes are classified to haploid in this panel as they reflect the haplotypes of female pronucleus. The mean numbers of crossovers are marked as dotted lines. **e** Resolution of crossover determination in female haploid cells. Accumulative percentage of crossover numbers is shown as red curve. **f** Crossover location density plots for all chromosomes in female and male mice, showing the crossover density by the distance from the centromere to telomere. **g** Average number of crossovers identified per chromosome per haploid cell. **h** The relationship between crossover frequency and chromosome size fitted with simple linear regression with a Pearson correlation of 0.80. **i** Distribution of inter-crossover distances measured in megabases of DNA length on all chromosomes. The red line represents the distribution curve fitting the experimental data. The black line shows the distribution of randomly generated distances. The dotted lines represent the median distances of the random distribution and experimental data distribution. **j** Merged distribution curves of inter-crossover distances measured in megabases of DNA length on all chromosomes in female and male meiosis. The dotted lines represent the median distances of the distribution curves. *P* value of Mann–Whitney *U* test = 0.0024. **k** Distribution of crossover classes along chromosome 1 in male and female haploid germ cells.

To study meiosis and recombination of female mice, we mated female B6D2F1 mice with DBA male mice to induce the extrusion of the PB2 and collect these haploid PB2 cells for sequencing (Fig. 7a). We also sequenced diploid PB1, MII oocytes, and zygotes containing a female pronucleus and a male pronucleus (DBA genome) (Fig. 7a). Parthenogenetic (PG) activation of mouse oocytes was also performed to acquire haploid genomes of oocytes (equivalent to female pronucleus in the zygote) without the incorporation of paternal genome (Fig. 7a). The number of different types of cells passed quality control was shown in Fig. 7b. The cells were sequenced at ~0.1–1.2× depth and the mean genome coverage was 10.4% (Fig. 7c). The mean coverage of diploid cells was 11.8%, a bit higher than that of haploid cells (9.6%) (Fig. 7c). A total of 15 aneuploid PB2 cells and 20 aneuploid PB1 cells were identified and verified with the advantage of Refresh-seq. The aneuploidy rates were 8.1% in PB2 cells and were 15.2% in PB1 of mice aged 4–8 weeks. The ratio of aneuploidy in female was much higher than that in male with elevated frequency of segregation errors, which was known not only in mice but also in humans^18,29^. It is probably due to prolonged arrest in the process of meiosis^51,52^, with enduring oxidative stress, accumulation of DNA damage, epigenetic changes and also maternal exposure to environmental stimuli^30^.

We then phased the genome of each cell and found crossovers of euploid cells. Note that the zygotes were obtained by mating female B6D2F1 mice (crossover visible) with DBA male mice (crossover invisible), and the female pronucleus from B6D2F1 contributed only one chromatid copy of each chromosome to the zygote^30^ with a complementary set of chromosomes contributed by the male pronucleus (DBA). We deduced the haplotypes of female pronucleus: the paternal alleles would show as homozygous and maternal alleles would show as ‘heterozygous’ due to the incorporation of DBA genome from the male pronucleus (Fig. 7a). Thus, the zygotes were classified to haploid in analysis of crossovers as they reflected the haplotypes of female pronucleus. We identified 2522 crossovers in 141 normal diploid cells, with an average of 18 crossovers per cell (Supplementary Fig. S7a). Of the 281 female normal haploid cells, a total of 3799 crossovers were identified, with an average of 14 recombination events per cell (Fig. 7d). The crossover numbers in diploid did not reach twice that in haploid cells as some of the false negatives in the detection of crossovers in diploid cells^29^. Crossover frequency in female haploid germ cells was 1.25 times that in sperm (13.5 vs 10.8), which was consistent with the results in C57BL/6J × CAST/EiJ F1 mice^19^. It might be attributed by ~20% longer chromosome axes in female mice than male^36^. Refresh-seq could detect the homologous crossover events of the paired cells. In the paired MII and PB1 shown in Supplementary Fig. S7b, c, all corresponding crossovers were located at the same regions within 1 to 3 Mb genomic distances. In the paired PG oocyte and PB2 shown in Supplementary Fig. S7d, e, two homologous crossovers at the same locations were observed in chromosomes 2 and 11, within a 10 kb genomic distance.

In order to compare homologous recombination in female and male mice, only haploid cells (PG oocytes and PB2) were included in the downstream analysis. Locations of crossovers in oocytes and PB2 were assigned to a median resolution of ~283 kb (Fig. 7e). About 42%, 30% and 15% of the crossovers could be assigned to intervals of 200 kb, 100 kb, and 30 kb, respectively. Thus, Refresh-seq could also identify crossovers in female germ cells with high resolution at relative low sequencing depth as well. The crossover location density plot of female mice showed fewer enrichments in centromere-proximal regions, which was the same as in male (Fig. 7f). The sub-telomeric regions showed milder enrichments of crossovers in female mice than male (Fig. 7f), consistent with previous results^53–55^.

Crossover frequency of each chromosome was calculated (Fig. 7g) and chromosome 1 featured the highest frequency of 1.016, which meant that each chromosome 1 in each haploid cell had one crossover on average, equivalent to two crossovers in a pair of chromosome 1 per meiosis. It was consistent with the longest chromosome axes of chromosome 1 in mice^56^. The recombination rate of chromosome 1 in female was 0.75 cM/Mb, 1.2 times higher than 0.64 cM/Mb in male mice, also agreeing with the results previously reported^16^. A nearly linear correlation between chromosome size and chromosome recombination frequency was also observed in female germ cells, with a Pearson correlation coefficient of 0.80 (Fig. 7h).

We then studied crossover interference in haploid germ cells (PG oocytes and PB2) in female mice. The average distance between two crossover events was 85 Mb in female for all chromosomes, longer than 54 Mb based on random simulation (Fig. 7i). Inter-crossover distance showed differences between female and male meiosis at the scale of genetic distances (number of base pairs), with oocytes and PB2 having weaker interference (85 Mb) compared with sperm (92 Mb) (Fig. 7j). It was consistent with the fact that oocytes had more crossovers than sperm. However, when we measured inter-crossover distance at the scale of the physical distances of the synaptonemal complex (SC) length (μm), the interference in both sexes was similar (Supplementary Fig. S7f). Consistent results were also found in chromosome 1 of mice by genetic linkage analyses^19^ and in humans by single-cell genome sequencing^29^. A maximum of three recombination crossover events were observed to occur simultaneously on chromosome 1 (Fig. 7k). No crossovers were found on chromosome 1 in 25% of the female haploid germ cells and 36% of sperm. Single crossovers were found on chromosome 1 in about 50% of germ cells of both sexes, ensuring one crossover per chromosome pair per meiosis to make sure proper segregation of homologous chromosomes^35^. Frequencies of multiple crossovers were different between sexes, with PB2 and oocytes having higher frequencies of double and triple crossovers on a chromosome 1 than sperm (Fig. 7k). The overall frequency was consistent with previous reports^19^.

Detection and phasing of SVs (insertions and deletions) were also performed in PG oocytes and PB2. A total of 34,916 SV events supported by the gold standard were detected in oocytes and PB2, including 20,106 deletions and 14,810 insertions. The length peak at 6–7 kb (LINE1 elements) was visible in deletions and peaks at 200 bp (B1 elements) were clear for both deletions and insertions (Supplementary Fig. S8a), as seen in sperm cells. At the level of over one read per cell, the precision of SVs supported by three cells was 83% and the recall was 49% (Supplementary Fig. S8b). We used SVs supported by over two reads per cell to perform SV phasing. Of the phased SVs, 96.7% were precisely phased to the true haplotype in the genome and the overall recall of true-phased SVs was 54.0% (Supplementary Fig. S8c, d and Table S1). The annotation of the correctly phased SVs in female haploid cells was performed (Supplementary Fig. S8e, f) and the overall distribution of different types of SVs was also similar with that in the bulk samples (Supplementary Fig. S6c, d). Thus, we validated for the first time that chromosome-wide hetSV phasing was also feasible with single-cell genome sequencing data from haploid female germ cells (PG oocytes and PB2).

## Discussion

We combine restriction endonuclease cutting and ligation strategy with TGS platform for single-cell genome sequencing for the first time and develop Refresh-seq, a single-cell long-read genome sequencing technique. Refresh-seq has increased genome coverage and uniformity compared with SMOOTH-seq. It has improved recovery rate for the two alleles of a diploid cell, even at a very shallow sequencing depth, having great potential for medical applications such as preimplantation genetic diagnosis^57^. This method is easy to be implemented and many samples can be processed manually without complicated automation. It’s also adjustable according to different restriction enzymes used to meet different demands. Refresh-seq is based on the TGS platform and can detect SVs as well as repeat elements inside the variant structures effectively. Refresh-seq has limitations as well. Due to the efficiency of ligation reaction, the lengths of amplicons only reach 2–3 kb, much shorter than SMOOTH-seq whose amplicon length is ~6 kb. Thus, Refresh-seq can not capture very long insertion events due to its amplicon length range.

The library construction of Refresh-seq can be accomplished within one day and the cost of library construction was $3.6 per cell for one-tube version of Refresh-seq and $2 per cell for multiplexed version. The cost can be even lower if Refresh-seq is to be combined with microfluidic bioprocessor capable for nanoliter dispensing. Microfluidic Refresh-seq may also have better performance in genome coverage because bulk samples started with 10 pg of genomic DNAs achieved only ~9% coverage with ~1× sequencing depth whereas Refresh-seq for single HG001 cells whose library construction was 1/10 volume of that in bulk achieved ~21.4% genome coverage. It may be that microfluidic Refresh-seq with much smaller volume (500- to 1000- fold less reagent volumes) can increase the relative concentration of genomic DNAs to a larger extent^58^ and improve ligation and PCR efficiency.

We have applied Refresh-seq to study meiosis of single germ cell in male and female B6D2F1 mice successfully. For sperm, PG oocytes and PB2 sequenced at ~0.1–0.3× depth, the mean coverage of sperm is about 5% and mean coverage of haploid PG oocytes and PB2 is 7.7%. It is consistent with coverage of sperm and oocytes amplified with MALBAC. The coverage of sperm is lower than oocytes probably due to the condensed chromatin structures of sperm^23,29^. We have acquired high-resolution genetic maps of male and female meiosis recombination at low sequencing depth and have revealed differences in female and male meiosis in mice using this new single-cell whole-genome sequencing method.

Refresh-seq works well in screening aneuploid sperm and oocytes due to its high uniformity and low allele dropout rate, making it promising in preimplantation genetic diagnosis. Due to its long read-length compared with NGS platforms, it also has advantage in detecting SVs especially in highly repetitive or low-complexity genomic regions. We have successfully performed chromosome-wide hetSV phasing with Refresh-seq data of sperm cells as well as female haploid germ cells respectively, and have analyzed the repeat element features in these SVs. Expansions of simple sequence repeats affect the nervous systems and cause diseases such as amyotrophic lateral sclerosis, Parkinson’s disease, Huntington disease, recessive ataxia and fragile X syndrome^59,60^, which largely affected human health. De novo mutations can also occur in families previously unaffected, thus it’s helpful to apply long-read whole-genome sequencing for preimplantation genetic diagnosis. Our Refresh-seq offers a new way to resolve these clinically relevant problems.

## Materials and methods

### Cell culture

NA24385 cells (HG002) and GM12878 Cells (HG001) were cultured in RPMI1640 (Gibco, cat# 11875093) with 15% fetal bovine serum (FBS, Gibco, cat# 26140079) and 1× Pen/Strep (Gibco, cat# 15140122) at 37 °C with 5% CO_2_. K562 cells were cultured in RPMI1640 with 10% FBS, 1× l-glutamine and 1× Pen/Strep. 3T3 cells were maintained in Dulbecco’s modified Eagle’s medium (DMEM)/high glucose (Corning, cat# 10-013-CV) with 10% FBS and 1× Pen/Strep. 3T3 cells were digested with 0.25% Trypsin-EDTA (Gibco, cat# 25200056) while K562 and GM12878 cell lines were harvested directly to prepare single cell suspensions.

GM12878 genomic DNA (gDNA) was extracted using the QIAGEN DNeasy Blood and Tissue Kit (QIAGEN, cat# 69504) following the manufacturer’s Quick-StartProtocol. The extracted GM12878 gDNA was quantified using the Equalbit 1× dsDNA HS Assay Kit (Vazyme, cat# EQ121).

### The isolation and FACS sorting of mice sperm

B6D2F1 mice were maintained on a 12/12-hour light/dark cycle and all animal experiments were performed under the guidelines of Ethical Committee for use of laboratory animals at Peking University. Mature mouse spermatozoa were obtained from epididymides of B6D2F1 mice using the swim-up assay^61^. Briefly, epididymis was pricked with a needle and placed into preheated HTF medium (Nanjing Aibei Biotechnology Co, cat# M1130) for 1 h at 37 °C. The top fractions containing motile sperm were collected and centrifuged. The cell pellets were resuspended with 0.1% phosphate-buffered saline-bovine serum albumin for three times. Cell suspension was incubated at 55 °C to inactivate the sperm and stained with DAPI and then sorted into 96-well plates by FACS.

### Collection of polar bodies

The 4- to 8-week-old female B6D2F1 mice were super-ovulated by intraperitoneal injection of 5 IU PMSG (pregnant mare serum gonadotropin, Sansheng Biological Technology, cat# 21958956) and then after 46 to 48 h 5 IU human chorionic gonadotrophin (hCG, Sansheng Biological Technology, cat# 110041282).

Then, the female mice were mated with DBA/2NCrl (DBA) male mice one to one. Females were sacrificed by cervical dislocation and embryos were collected at 20–24 h post hCG from the oviducts of female mice. Cumulus cells were removed by treating with 300 µg/mL hyaluronidase (Sigma, cat# H4272) in M2 media (Nanjing Aibei Biotechnology Co, cat# M1250) with gentle pipetting. Embryos were depleted of zona pellucida with Tyrode’s solution (Sigma, T1788) and quickly transferred to 1:1 Accutase (Sigma, cat# A6964):0.25% Trypsin-EDTA (Gibco, cat# 25200056) preheated to 37 °C. The embryos were pipetted up and down by microcapillary to dissociate the PB1 and PB2 from zygotes.

Artificial activation of mouse oocytes with SrCl_2_ was performed to acquire maternal genomes without the incorporation of paternal genomes^62^ and the protocol was previously optimized^63,64^. Briefly, embryos were exposed to 10 mM concentrations of SrCl_2_ (Sigma, cat# 439665) for 3 h in 20 μL Ca^2+^ free CZB medium droplet covered by mineral oil (Sigma, M8410) in 37 °C incubator filled with 5% CO_2_. The dissociation of PB1, PB2 as well as zygotes was same as described above.

### Library construction of Refresh-seq

The detailed protocols for Refresh-seq and Refresh-seq (multiplexed) are available in Supplementary information.

Individual cells were collected with a microcapillary connected to a mouth pipette or by FACS after washed with 0.1% phosphate-buffered saline-bovine serum albumin for three times. Each single cell was placed into a 0.2 mL thin-walled PCR tube containing 2.5 μL lysis buffer. The cells were lysed at 50 °C for 3 h to digest histones and then 70 °C for 30 min to inactivate the protease.

For Refresh-seq, after single cell lysis, restriction digestion of single cell gDNA was performed by adding 0.5 μL 10× buffer, 1.9 μL water and 0.1 μL restriction enzyme. The reaction program was adjustable according to the restriction enzyme used. For *Eco*RI (NEW ENGLAND BioLabs, cat# R3101L) and *Sac*I (NEW ENGLAND BioLabs, cat# R3156S), the restriction digestion was performed at 37 °C 15 min, and then 65 °C 20 min to inactivate the restriction enzyme. For *Asi*SI (NEW ENGLAND BioLabs, cat# R0630S), the restriction digestion was performed at 37 °C 1 h, and then 80 °C 20 min to inactivate the restriction enzyme.

Then end repair and dA-tailing (Kapa Biosystems, KAPA HyperPrep kit, cat# KK8504) were performed and dsDNA adapters (NEBNext Singleplex Oligos for Illumina) were ligated to 3’-dA-tailed molecules, after which USER Enzyme (uracil-specific excision reagent, NEW ENGLAND BioLabs, cat# M5505L) was added to the ligation mixture. Each sample was purified with 1 volume of AMPure XP beads (BECKMAN COULTER, cat# A63882) and amplified with Barcode-P5 (GCTA-[24 bp P5-barcode 81-96]-TACACTCTTTCCCTACACGACGCTCTTCCGATCT) and Barcode-P3 (ATCG-[24 bp P3-barcode 1-24]-GACTGGAGTTCAGACGTGTGCT) (Supplementary Table S2). The PCR program was 98 °C 45 s, 98 °C 15 s, and then 20 cycles of 98 °C for 15 s, 65 °C for 30 s, and 72 °C for 5 min. After that, gDNA amplicons were purified with 0.7× AMPure XP beads for twice (0.65× AMPure XP beads twice for haploid cells). The purified amplicons were quantified using Equalbit 1× dsDNA HS Assay Kit.

For Refresh-seq (multiplexed), single-stranded oligos were annealed with their appropriate partner before ligation. Synthesized oligos (NEB same-A: GATCGGAAGAGCACACGTCTGAACTCCAGTC with 5’Phospholation modification and Barcoded-B: ACACTCTTTCCCTACACGAC-[24 bp adaptor-barcode 31-46]-GCTCTTCCGATC*T) (Supplementary Table S2) were dissolved to an initial concentration of 100 μM. To create a duplex adapter, NEB same-A and Barcoded-B were combined in a 1:1 ratio for a total annealed adapter concentration of 50 μM (use 50 μL adapter NEB same-A and 50 μL adapter Barcoded-B) and mixed thoroughly. Barcoded adaptors were ligated to the fragments after the process of end repair and dA-tailing. Cells with different barcodes were pooled together and purified with 1 volume of AMPure XP beads, after which library amplification was performed with Common-P5 (ACACTCTTTCCCTACACGAC) and Barcode-P3 (ATCG-[24 bp P3-barcode 1-24]-GACTGGAGTTCAGACGTGTGCT) (Supplementary Table S2). The PCR program and the purification afterwards were the same with Refresh-seq.

Each library was loaded into R9 flow cell and sequenced on PromethION HAC (high accuracy) model.

### Basic processing of Refresh-seq data

The raw data produced by ONT sequencing was converted into fastq format. We used nanoplexer v0.1 (https://github.com/hanyue36/nanoplexer) to demultiplex single cells from the noisy long-read library for successive two times according to our library structure of dual single-cell barcodes. Cutadapt v3.4 (https://github.com/marcelm/cutadapt) was applied to remove adaptors at 5’ ends and 3’ ends in demultiplexed reads as well as filtering out reads shorter than 500 bp to preserve long reads only. The adaptor sequences were shown above in the library construction. Trimmed reads were aligned to the reference genome hg38 or mm10 by minimap2 v2.24 (https://github.com/lh3/minimap2). We used samtools v1.14 (https://github.com/samtools/samtools) to filter out the reads mapping quality less than 30 and remove PCR duplicates in each single cell.

### Assessment of uniformity of Refresh-seq data

Since single-cell sequencing libraries were always inherently noisy and amplification bias affected the subsequent analysis, we need to know the uniformity of our data. To compare the amplification uniformity of different methods, we used three approaches to describe it.

We used Spikiness $(s=\frac{{\sum }_{t=1}^{T-1}\left|{x}_{t+1}-{x}_{t}\right|}{{\sum }_{t=1}^{T}{x}_{t}}$) and Shannon entropy ($e=\frac{-{\sum }_{t=1}^{T}{x}_{t}}{X{\cdot }\log \left(\frac{{x}_{t}}{X}\right)}$ and $X={\sum }_{t=1}^{T}{x}_{t}$) to assess library quality. Among which Spikiness was a measure for the bin-to-bin variation of the read count and Shannon entropy was a measure of the uniformity of the read distribution. The third strategy used coefficient of variation to measure the dispersion of the read distribution with different size of windows. Most importantly, blacklist regions were out of consideration which could have a huge impact on the results so as to cover the real situation.

### Evaluation of cross-contamination

To evaluate cross-contamination among single cells, we used the strategy mapping human-mouse mixed reads to the reference genome mixed by hg38 and mm10 and other steps were the same as data pre-processing. It was worth mentioning that the mixed genome was indexed by minimap2 with the parameter ‘-I 10 G’. Only high-quality single cells were used.

Then we counted the read numbers for mm10 and hg38 alignments apart for each single cell. The species of reference genome majorly mapped was determined to be the species of this single cell. Cells with high proportion of reads mapping to minor genome more than 10% were identified as cross-contaminates or doublets.

### CNV analysis

We used Control-FREEC v11.6 (https://github.com/BoevaLab/FREEC) to detect CNVs of K562 cells and B6D2F1 germ cells. The window was set to 1 Mb, and the ploidy was set to 3 for K562 cells and 1 for B6D2F1 haploid germ cells. We calculated the mean ratio of reads detected in each window and normalized to 3 for K562 and 1 for B6D2F1 haploid germ cells as the average copy number across the whole genome. The fold change of copy number in each window against the average copy number was considered as CNV. To evaluate the deviation of profiling CNVs, we calculated the CV for each cell with the mean copy number of single cells as the baseline.

### Identification and validation of SNPs

On account of the open access to a benchmark SNP calling set of the HG002 and HG001 genome on GIAB, we used these diploid cell lines as objects of study to evaluate the advantages of our method in the aspect of amplifying two alleles in a cell.

To evaluate the precision at known SNP site in our data, we used longshot v.0.4.5 (https://github.com/pjedge/longshot) to call SNVs. Considering that our data was at low sequencing depth, we set minimum coverage (of reads passing filters) to consider position as a potential SNV to 2 in order to include as many SNPs as possible. We evaluated the precision of homozygous SNP sites among benchmark SNP calling set on longshot outputs. Only SNVs having the same mutation as benchmark were regarded as true positive.

To evaluate the precision of SNP phasing, we used R package Hapi (https://github.com/rli012/Hapi/) which is a novel easy-to-use and high-efficient algorithm that only requires 3 to 5 gametes to reconstruct accurate and high-resolution haplotypes of an individual. We constructed a matrix with the information of genotype data of known hetSNPs in each gamete cell as Hapi input. It reported the high-resolution haplotypes as well as the confidence level of each phased hetSNPs. The phased hetSNPs consistent with parental hetSNPs were considered as the true positive and we calculated the precision.

### Evaluation of heterozygosity of SNP sites

To genotype our data, we used whatshap v.1.5 (https://whatshap.readthedocs.io/en/latest/) to compute genotype likelihoods for all three genotypes (0/0, 0/1, 1/1) at given variant positions and output them in a VCF file together with a genotype prediction. We ran it using the command ‘whatshap genotype --reference ref.fasta -o genotyped.vcf variants.vcf reads.bam’. The file variants.vcf was the HG002 or HG001 SNP benchmark downloaded from GIAB or B6D2F1 bulk SNP benchmark. We calculated the proportion of heterozygotes as the ratio of two alleles detected.

### Crossover event calling

To identify the most likely sequence of haplotypes, we used an HMM to correct the genotyping errors in read calls. Before genotyping, we need a benchmark hetSNP set. Genomic reads of the C57 and DBA samples sequenced on the Illumina sequencing platform from bulk library were used to call high-confident SNPs and formed a benchmark SNP set. Only hetSNPs were filtered for downstream analysis.

To infer the genome positions of crossovers in each sperm, we did the following:

Based on the benchmark SNP set, we used ‘whatshap genotype’ command to score every hetSNP loci and gave out the most likelihood genotype at the SNP sites at first. Only homozygous (0/0 or 1/1) genotype locations were reserved because our sperm sample was expected to be haploid and a heterozygous genotype is likely due to sequencing or mapping errors. This step output a file recording the observational genotype for HMM algorithm.

Due to the existence of crossovers, each sperm cell was expected to be composed of segments of DBA and C57 genomes which represented the underlying hidden states we wished to infer. We introduced 0 and 1 to signify the hidden states which inherited from C57 and DBA genome and we set initial probabilities of the two states equal to 0.5 since both haplotypes were indiscriminate initially. On account of sequencing and alignment errors in addition to other uncertainty factors, we set P(h = 1|s = 1) = P(h = 0|s = 0) = 0.99 and P(h = 0|s = 1) = P(h = 1|s = 0) = 0.01 as the emission probabilities of emitting an observed state after the hidden state is determined. The transition of states between two adjacent sites reflected a crossover event from C57 to DBA or from DBA to C57. So we set the transition probability between linked sites to be P(s_i+1_ = 0|s_i_ = 1) = P(s_i+1_ = 1|s_i_ = 0) = 0.2. Then we ran the HMM in both the forward-chromosomal and the reverse-chromosomal directions and kept the sites with same hidden state in both directions left. Boundaries of the last SNP in the first haplotype and the first in the next were defined as crossover regions.

Due to the existence of random DBS repair and low accuracy of nanopore sequencing platform (sequencing accuracy of 96.5%), we can see many “crossovers” within a few SNPs. By reasons of the low accuracy of TGS sequencing data and low depth of our data, these crossovers supported by less than 100 SNPs or spaced shorter than 0.5 Mb seemed more likely to be pseudo. Thus, we filtered out these regions and finally determined the crossovers every chromosome in each cell.

To plot the density of the distribution of crossover regions, we calculated the counts of all crossover events across all samples in 1 Mb windows on each chromosome. We used ‘bedtools makewindows’ to divide each chromosome into 1 Mb windows with the parameter ‘-w 1,000,000’.

### Filtering out of diploid cells from single sperm Refresh-seq dataset

In diploid cells or doublets which contained two haplotypes, fragments from paternal and maternal were randomly distributed and were not able to maintain a state in a large genome region due to allele dropout, and thus we detected consecutively observed SNP alleles that appeared on different parental haplotypes and defined it as ‘non-continuity score’. To filter out diploid cells whose non-continuity scores, we summed the non-continuity scores of all autosomes except for the autosome with the highest scores to avoid mistakenly identifying cells with chromosome gains as diploid cells. This resulted in a clear inflection point wherein doublets had a high non-continuity score. All cells below this inflection point (identified with the function ‘ede’ from the R package ‘inflection’ (https://CRAN.R-project.org/package=inflection) were labeled as haploid cells.

### Aneuploidy and chromosome arm loss/gain

As described in CNV analysis, we ran control-freec with parameter ‘ploidy = 1’ and ‘window = 1,000,000’ to identify copy number variations for the cells labeled as haploid. The cells with copy number reduction meanwhile this region having small quantity of SNPs compared to other normal chromosomes were considered as chromosome deficiency. The cells with copy number increase meanwhile this region showing high heterozygosity and high non-continuity scores were considered as gain of chromosomes.

### SV calling and benchmarking

SV identification for each single cell was performed by cuteSV v1.0.10 (https://github.com/tjiangHIT/cuteSV), a sensitive, fast and scalable long-read based SV detection approach suitable for nanopore long reads. We set the recommended parameters for nanopore as ‘--max_cluster_bias_INS 100 --diff_ratio_merging_INS 0.3 --max_cluster_bias_DEL 100 --diff_ratio_merging_DEL 0.3’ to process single-cell bam files. We also set ‘--min_support 1’ to reach resolution at single cell level.

We integrated SV calls from all B6D2F1 sperm cells passed quality control using SURVIVOR V1.0.7 (https://github.com/fritzsedlazeck/SURVIVOR) with ’SURVIVOR merge’ command. The maximum breakpoints distance to merge two SVs was set to 500 and the minimum size of the reserved SV was 50 bp, besides, the SVs type and strand type was taken into account. By changing the number of cells supported, we got each SV supported by at least one to ten cells.

Before evaluation of SV calling, we need to get a benchmark SV set. Genomic reads of the C57 and DBA bulk samples sequenced on the ONT platform were used to produce the high-confidence benchmark SV set.

To compare the SVs identified with the benchmark SV set, we used the following criteria. For insertions, the breakpoints need to be within ±500 bp of each other. For deletions, the two SV regions need to be overlapped. Meanwhile, the length of both insertion and deletion events should be consistent with benchmark SV set with 100 bp tolerance range. Then we used precision (Precision = true positive/(true positive + false positive)) to measure the ratio of true positives in all detected positives in order to quantify the performance of SV detection.

### SV phasing

To phase SVs, we constructed a sparse matrix containing the information about the existence or not of each SVs each cell based on benchmark SV set. That was, 0 represents C57 genotype and 1 represents DBA genotype. The conditions for determining whether an SV exists were its length should not differ from benchmark by 100 bp and the breakpoints need to be within ±100 bp of each other. If a site was covered by reads but no SV was detected, the site was labeled as the other genotype. The core algorithm to phase SVs in a chromosome consists of three main steps: (1) data pre-processing; (2) draft haplotype inference; (3) haplotype assembly. For low coverage sequencing, heterozygous SVs that were genotyped in at least five sperm cells can be selected to form a ‘precursor’ framework for the draft haplotype construction using the ‘hapiFrameSelection’ function (https://github.com/rli012/Hapi/). Then we selected the top 100 cells with the highest total number of SVs to form a pre-phasing framework and removed SVs supported by less than 3 cells. To improve phasing accuracy, we corrected the SVs which seemed more likely to be an error from technical limitations or DSB repairs. The proofreading strategy followed the steps below: First, we selected one cell as a test, and ran the HMM in the other cells with transition probability between linked sites being *P* = $1-{e}^{-d\times {10}^{-8}}$ where *d* represented the distance. Then we checked if the rest 99 cells had the same SV genotypes as the test. SVs supported by more than 5 even half of cells inconsistent with test were regarded as errors in the test. Each cell did the same steps above in turn. We considered that SVs with more than 5 cells labeled as wrong were error-prone and excluded from subsequent phasing, but SVs with less than 3 cells labeled as wrong were corrected using the ‘flipFun’ function from Hapi R package in errant cells as they looked more likely random mistakes. At this point, the basic framework was constructed. Missing genotypes in each cell were iteratively imputed by observed genotypes in other cells to facilitate the draft haplotype inference using function ‘imputationFun1’ with parameter ‘nSPT=2’ which meant several successive heterozygous SVs in a sperm cell can be imputed only if imputations were supported by more than 2 consecutive SVs with consistent genotype and no imputation conflict from different supporting cells. We kept SVs less than 2 missing genotypes and cells without missing genotypes to run ‘hapiPhase’ function. The preliminary phasing results were proofread using ‘hapiBlockMPR’ function with parameter ‘cvlink=2’ and ‘smallBlock=2’. Finally, we used function ‘hapiAssemble’ to assemble the consensus high-resolution haplotypes.

### Supplementary information

Supplementary information

## Data Availability

Data sequenced from human cell lines are available in National Center for Biotechnology Information (NCBI). Refresh-seq data for human cell lines was deposited in Sequence Read Archive (SRA, PRJNA971770). Refresh-seq data for mice germ cells and mice cell line have been deposited in the Genome Sequence Archive^65^, in National Genomics Data Center^66^, China National Center for Bioinformation/Beijing Institute of Genomics, Chinese Academy of Sciences (GSA: PRJCA016905) that are publicly accessible at https://ngdc.cncb.ac.cn/gsa. Data of SMOOTH-seq were from the work of Xie et al.^20^ and publicly available through SRA (PRJNA800164).

## References

[1] Huang L, Ma F, Chapman A, Lu S, Xie XS (2015). Single-cell whole-genome amplification and sequencing: methodology and applications. Annu. Rev. Genomics Hum. Genet..

[2] Telenius H (1992). Degenerate oligonucleotide-primed PCR: general amplification of target DNA by a single degenerate primer. Genomics.

[3] Dean FB (2002). Comprehensive human genome amplification using multiple displacement amplification. Proc. Natl. Acad. Sci. USA.

[4] Zong C, Lu S, Chapman AR, Xie XS (2012). Genome-wide detection of single-nucleotide and copy-number variations of a single human cell. Science.

[5] Fu Y (2015). Uniform and accurate single-cell sequencing based on emulsion whole-genome amplification. Proc. Natl. Acad. Sci. USA.

[6] Chen C (2017). Single-cell whole-genome analyses by linear amplification via transposon insertion (LIANTI). Science.

[7] Gonzalez-Pena V (2021). Accurate genomic variant detection in single cells with primary template-directed amplification. Proc. Natl. Acad. Sci. USA.

[8] Xing D, Tan L, Chang CH, Li H, Xie XS (2021). Accurate SNV detection in single cells by transposon-based whole-genome amplification of complementary strands. Proc. Natl. Acad. Sci. USA.

[9] Macintyre G, Ylstra B, Brenton JD (2016). Sequencing structural variants in cancer for precision therapeutics. Trends Genet..

[10] Ciriello G (2013). Emerging landscape of oncogenic signatures across human cancers. Nat. Genet..

[11] Fan X (2021). SMOOTH-seq: single-cell genome sequencing of human cells on a third-generation sequencing platform. Genome Biol..

[12] Di Felice F, Micheli G, Camilloni G (2019). Restriction enzymes and their use in molecular biology: an overview. J. Biosci..

[13] Gu H (2021). Smart-RRBS for single-cell methylome and transcriptome analysis. Nat. Protoc..

[14] Davey JW, Blaxter ML (2011). RADSeq: next-generation population genetics. Brief. Funct. Genomics.

[15] Xie C (2022). Meiotic recombination: insights into its mechanisms and its role in human reproduction with a special focus on non-obstructive azoospermia. Hum. Reprod. Update.

[16] Lichten M (2008). The recombinational anatomy of a mouse chromosome. PLoS Genet..

[17] Myers S (2006). The distribution and causes of meiotic recombination in the human genome. Biochem. Soc. Trans..

[18] Hua R, Liu M (2021). Sexual dimorphism in mouse meiosis. Front. Cell Dev. Biol..

[19] Petkov PM, Broman KW, Szatkiewicz JP, Paigen K (2007). Crossover interference underlies sex differences in recombination rates. Trends Genet..

[20] Xie H (2022). De novo assembly of human genome at single-cell levels. Nucleic Acids Res..

[21] Bakker B (2016). Single-cell sequencing reveals karyotype heterogeneity in murine and human malignancies. Genome Biol..

[22] Andrews KR, Good JM, Miller MR, Luikart G, Hohenlohe PA (2016). Harnessing the power of RADseq for ecological and evolutionary genomics. Nat. Rev. Genet..

[23] Lu S (2012). Probing meiotic recombination and aneuploidy of single sperm cells. Science.

[24] Fan HC, Wang J, Potanina A, Quake SR (2011). Whole-genome molecular haplotyping of single cells. Nat. Biotechnol..

[25] Lyu R (2022). sgcocaller and comapr: personalised haplotype assembly and comparative crossover map analysis using single-gamete sequencing data. Nucleic Acids Res..

[26] Bell AD (2020). Insights into variation in meiosis from 31,228 human sperm genomes. Nature.

[27] Weaver KJ (2022). A method for low-coverage single-gamete sequence analysis demonstrates adherence to Mendel’s first law across a large sample of human sperm. eLife.

[28] Schneider L, Tripathi A (2021). Progress and challenges in laboratory-based diagnostic and screening approaches for aneuploidy detection during pregnancy. SLAS Technol..

[29] Hou Y (2013). Genome analyses of single human oocytes. Cell.

[30] Charalambous C, Webster A, Schuh M (2023). Aneuploidy in mammalian oocytes and the impact of maternal ageing. Nat. Rev. Mol. Cell Biol..

[31] Hinch AG (2019). Factors influencing meiotic recombination revealed by whole-genome sequencing of single sperm. Science.

[32] Li R (2019). A high-resolution map of non-crossover events reveals impacts of genetic diversity on mammalian meiotic recombination. Nat. Commun..

[33] Chuang Y-C, Smith GR (2023). Meiotic crossover interference: methods of analysis and mechanisms of action. Curr. Top. Dev. Biol..

[34] Pratto F (2021). Meiotic recombination mirrors patterns of germline replication in mice and humans. Cell.

[35] Wooldridge LK, Dumont BL, Falush D (2023). Rapid evolution of the fine-scale recombination landscape in wild house mouse (*Mus musculus*) populations. Mol. Biol. Evol..

[36] Zhang L (2021). Crossover patterns under meiotic chromosome program. Asian. J. Androl..

[37] Ruiz-Herrera A (2017). Recombination correlates with synaptonemal complex length and chromatin loop size in bovids—insights into mammalian meiotic chromosomal organization. Chromosoma.

[38] Lynn A (2002). Covariation of synaptonemal complex length and mammalian meiotic exchange rates. Science.

[39] Weckselblatt B, Rudd MK (2015). Human structural variation: mechanisms of chromosome rearrangements. Trends Genet..

[40] Logsdon GA, Vollger MR, Eichler EE (2020). Long-read human genome sequencing and its applications. Nat. Rev. Genet..

[41] Wong JH (2019). Identification of intermediate-sized deletions and inference of their impact on gene expression in a human population. Genome Med..

[42] Lu JY (2021). Homotypic clustering of L1 and B1/Alu repeats compartmentalizes the 3D genome. Cell Res..

[43] Umylny B, Presting G, Efird JT, Klimovitsky BI, Ward WS (2007). Most human Alu and murine B1 repeats are unique. J. Cell Biochem..

[44] Zhou Y, Leung AW-S, Ahmed SS, Lam T-W, Luo R (2022). Duet: SNP-assisted structural variant calling and phasing using Oxford nanopore sequencing. BMC Bioinforma..

[45] Browning BL, Tian X, Zhou Y, Browning SR (2021). Fast two-stage phasing of large-scale sequence data. Am. J. Hum. Genet..

[46] Talsania K (2022). Structural variant analysis of a cancer reference cell line sample using multiple sequencing technologies. Genome Biol..

[47] Jakubosky D (2020). Discovery and quality analysis of a comprehensive set of structural variants and short tandem repeats. Nat. Commun..

[48] Morelli MA, Cohen PE (2005). Not all germ cells are created equal: aspects of sexual dimorphism in mammalian meiosis. Reproduction.

[49] Wang X, Pepling ME (2021). Regulation of meiotic prophase one in mammalian oocytes. Front. Cell Dev. Biol..

[50] Bolcun-Filas E, Handel MA (2018). Meiosis: the chromosomal foundation of reproduction. Biol. Reprod..

[51] MacLennan M, Crichton JH, Playfoot CJ, Adams IR (2015). Oocyte development, meiosis and aneuploidy. Semin. Cell Dev. Biol..

[52] Lodge C, Herbert M (2020). Oocyte aneuploidy-more tools to tackle an old problem. Proc. Natl. Acad. Sci. USA.

[53] Badge RM, Yardley J, Jeffreys AJ, Armour JA (2000). Crossover breakpoint mapping identifies a subtelomeric hotspot for male meiotic recombination. Hum. Mol. Genet..

[54] Froenicke L, Anderson LK, Wienberg J, Ashley T (2002). Male mouse recombination maps for each autosome identified by chromosome painting. Am. J. Hum. Genet..

[55] Brick K (2018). Extensive sex differences at the initiation of genetic recombination. Nature.

[56] Kelmenson PM (2005). A torrid zone on mouse chromosome 1 containing a cluster of recombinational hotspots. Genetics.

[57] Piyamongkol W (2003). Detailed investigation of factors influencing amplification efficiency and allele drop-out in single cell PCR: implications for preimplantation genetic diagnosis. Mol. Hum. Reprod..

[58] Hanlon VCT (2022). Construction of Strand-seq libraries in open nanoliter arrays. Cell. Rep. Methods.

[59] Paulson H (2018). Repeat expansion diseases. Handb. Clin. Neurol..

[60] Pascarella G (2022). Recombination of repeat elements generates somatic complexity in human genomes. Cell.

[61] Brykczynska U (2010). Repressive and active histone methylation mark distinct promoters in human and mouse spermatozoa. Nat. Struct. Mol. Biol..

[62] Paffoni A, Brevini TAL, Gandolfi F, Ragni G (2008). Parthenogenetic activation: biology and applications in the ART laboratory. Placenta.

[63] Ma S-F (2005). Parthenogenetic activation of mouse oocytes by strontium chloride: a search for the best conditions. Theriogenology.

[64] Ahmad AM (2021). Artificial activation of mouse oocytes with SrCl2 with minimal detrimental effect on early embryonic development. Pak. J. Zool..

[65] Chen, T. et al. The Genome Sequence Archive family: toward explosive data growth and diverse data types. *Genomics Proteomics Bioinformatics* **4**, 578–583 (2021). 10.1016/j.gpb.2021.08.001 PMC9039563 34400360

[66] Database Resources of the National Genomics Data Center, China National Center for Bioinformation in 2023. (2023). Nucleic Acids Res..

