# Supplementary material for: Mapping crossover events of mouse meiotic recombination by restriction fragment ligation-based Refresh-seq
Source: Cell Discov. 2024 Mar 5;10:26. doi: 10.1038/s41421-023-00638-9 (PMC10915157; doi:10.1038/s41421-023-00638-9)
Supplement: Supplementary file 1 — Supplementary information [file 41421_2023_638_MOESM1_ESM.pdf]

Supplementary Information for

Mapping crossover events of mouse meiotic recombination by restriction fragment ligation-based Refresh-seq

Yan Wang[1,2], Yijun Chen[1,2,3], Junpeng Gao[1,2,4], Haoling Xie[1,2], Yuqing Guo[1,2], Jingwei Yang[1,2], Jun'e Liu[1,2], Zonggui Chen[1,2,5], Qingqing Li[1,2], Mengyao Li[1,2], Jie Ren[1,2], Lu Wen[1,2] and Fuchou Tang[1,2,3,5]

[1]Biomedical Pioneering Innovation Center, School of Life Sciences, Peking University, Beijing, China.

[2]Beijing Advanced Innovation Center for Genomics (ICG), Ministry of Education Key Laboratory of Cell Proliferation and Differentiation, Beijing, China.

[3]Peking-Tsinghua Center for Life Sciences, Academy for Advanced Interdisciplinary Studies, Peking University, Beijing, China.

[4]Emergency Center, Zhongnan Hospital of Wuhan University, Wuhan, Hubei, China.

[5]Changping Laboratory, Beijing, China.

These authors contributed equally: Yan Wang, Yijun Chen

Correspondence: Fuchou Tang (tangfuchou@pku.edu.cn)

This file includes:

Supplementary Figs. S1 to S9

Supplementary Methods

Supplementary Tables S1 to S2

Supplementary Fig. S1

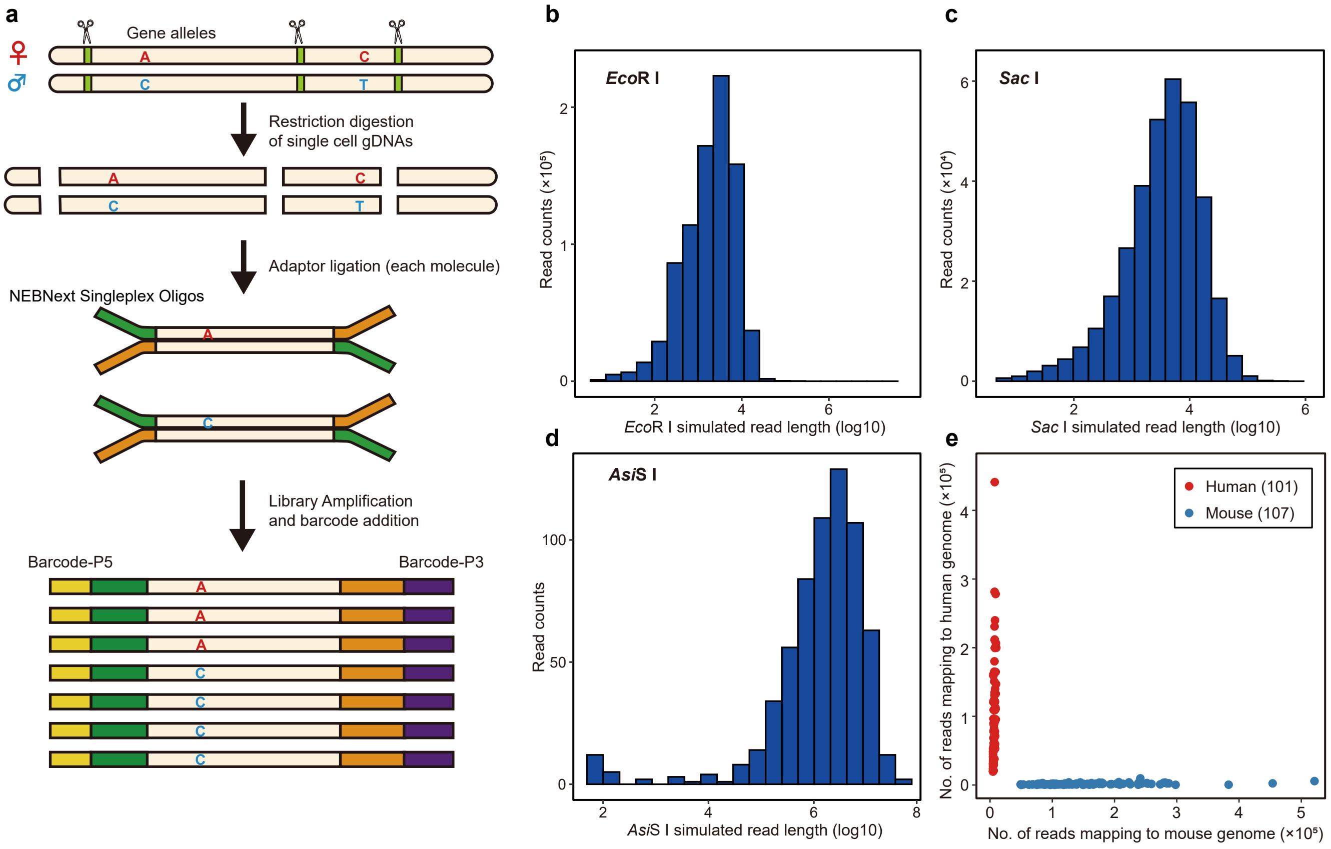

Supplementary Fig. S1 The schematic of Refresh-seq technique. a Schematic of single tube version of Refresh-seq. **b** Restriction fragment length simulation for *Eco*R I. **c** Restriction fragment length simulation for *Sac* I. **d** Restriction fragment length simulation for *Asi*S I. **e** Experiments for testing cross-contamination of Refresh-seq (multiplexed) on the human-mouse mixing cell lines.

Supplementary Fig. S2

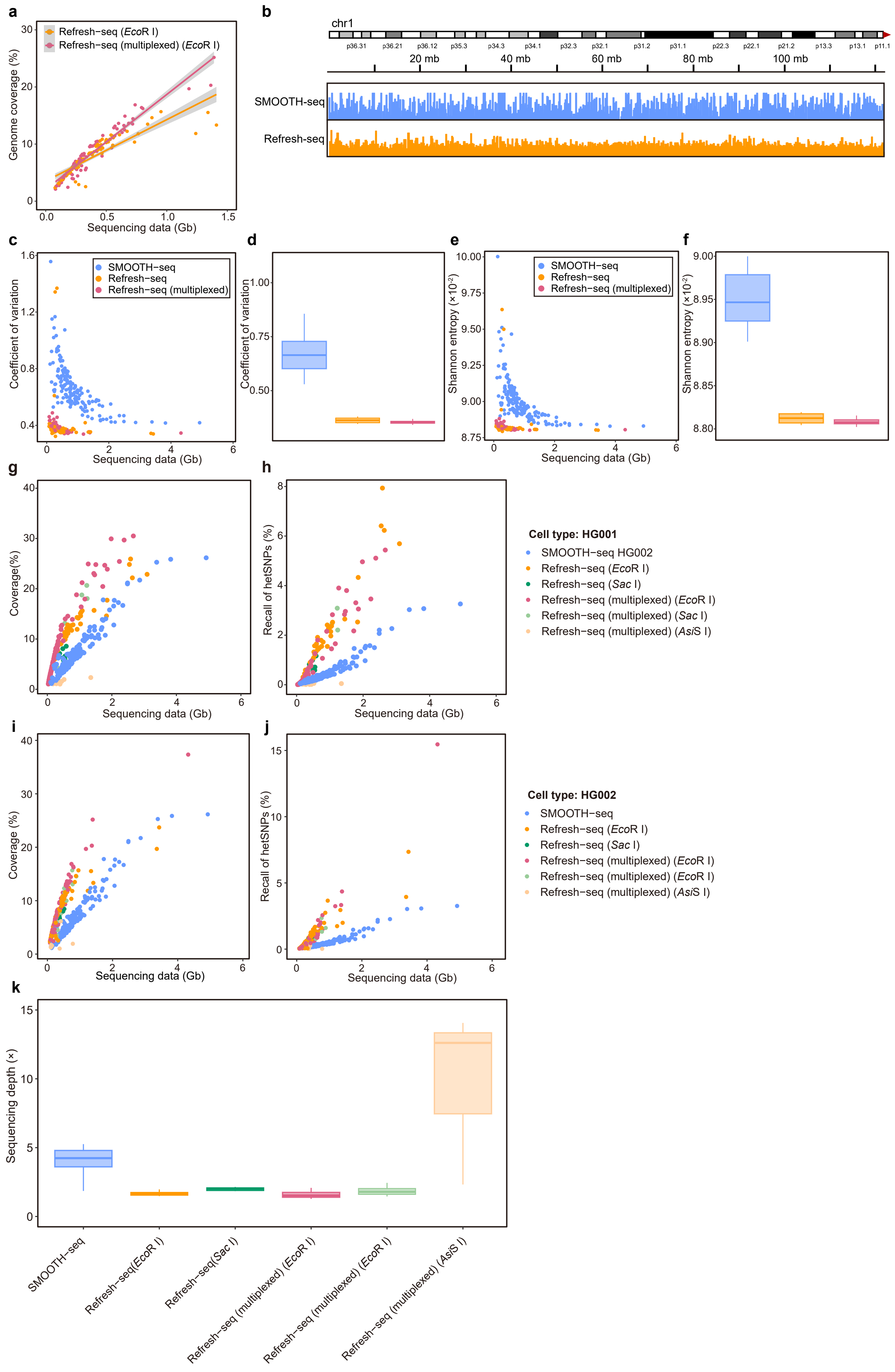

Supplementary Fig. S2 Comparison of Refresh-seq with SMOOTH-seq. a Scatter plot showing the sequencing data and genome coverage of Refresh-seq and Refresh-seq (multiplexed) performed on HG002 cells. Linear fitting of coverage and sequencing data is shown in lines and 95% credible intervals are shown as shading. **b** An Integrative Genomics Viewer illustration of the read distribution of SMOOTH-seq and Refresh-seq with the same sequencing depth. Raw reads on long arm of chromosome 1 are shown from single cell amplified with SMOOTH-seq and Refresh-seq. **c** Scatter plot showing the sequencing data and CV of each HG002 cell amplified with SMOOTH-seq and Refresh-seq and Refresh-seq (multiplexed). **d** Quantification of CVs shown in Supplementary Fig. S2c at ~0.25× sequencing depth. **e** Scatter plot showing the sequencing data and Shannon entropy of each HG002 cell amplified with SMOOTH-seq and Refresh-seq and Refresh-seq (multiplexed). **f** Quantification of Shannon entropy shown in Supplementary Fig. S2e at ~0.25× sequencing depth. **g** Scatter plot showing the sequencing data and genome coverage of each HG001 cell amplified with Refresh-seq (*Eco*R I/*Sac* I) and Refresh-seq (multiplexed) (*Eco*R I/*Sac* I/*Asi*S I). Data of SMOOTH-seq were from the HG002 cell line. **h** Scatter plot showing the sequencing data and the rates of detection of both alleles at hetSNP sites of each HG001 cell amplified with Refresh-seq (*Eco*R I/*Sac* I) and Refresh-seq (multiplexed) (*Eco*R I/*Sac* I/*Asi*S I). Data of SMOOTH-seq were from the HG002 cell line. **i** Scatter plot showing the sequencing data and genome coverage of each HG002 cell amplified with SMOOTH-seq, Refresh-seq (*Eco*R I/*Sac* I) and Refresh-seq (multiplexed) (*Eco*R I/*Sac* I/*Asi*S I). **j** Scatter plot showing the sequencing data and the rates of detection of both alleles at hetSNP sites of each HG002 cell amplified with SMOOTH-seq, Refresh-seq (*Eco*R I/*Sac* I) and Refresh-seq (multiplexed) (*Eco*R I/*Sac* I/*Asi*S I). **k** Sequencing depth of SMOOTH-seq, Refresh-

seq and Refresh-seq (multiplexed) using different restriction enzymes (*Eco*R I/*Sac* I/*Asi*S I) for HG002 cells.

Supplementary Fig. S3

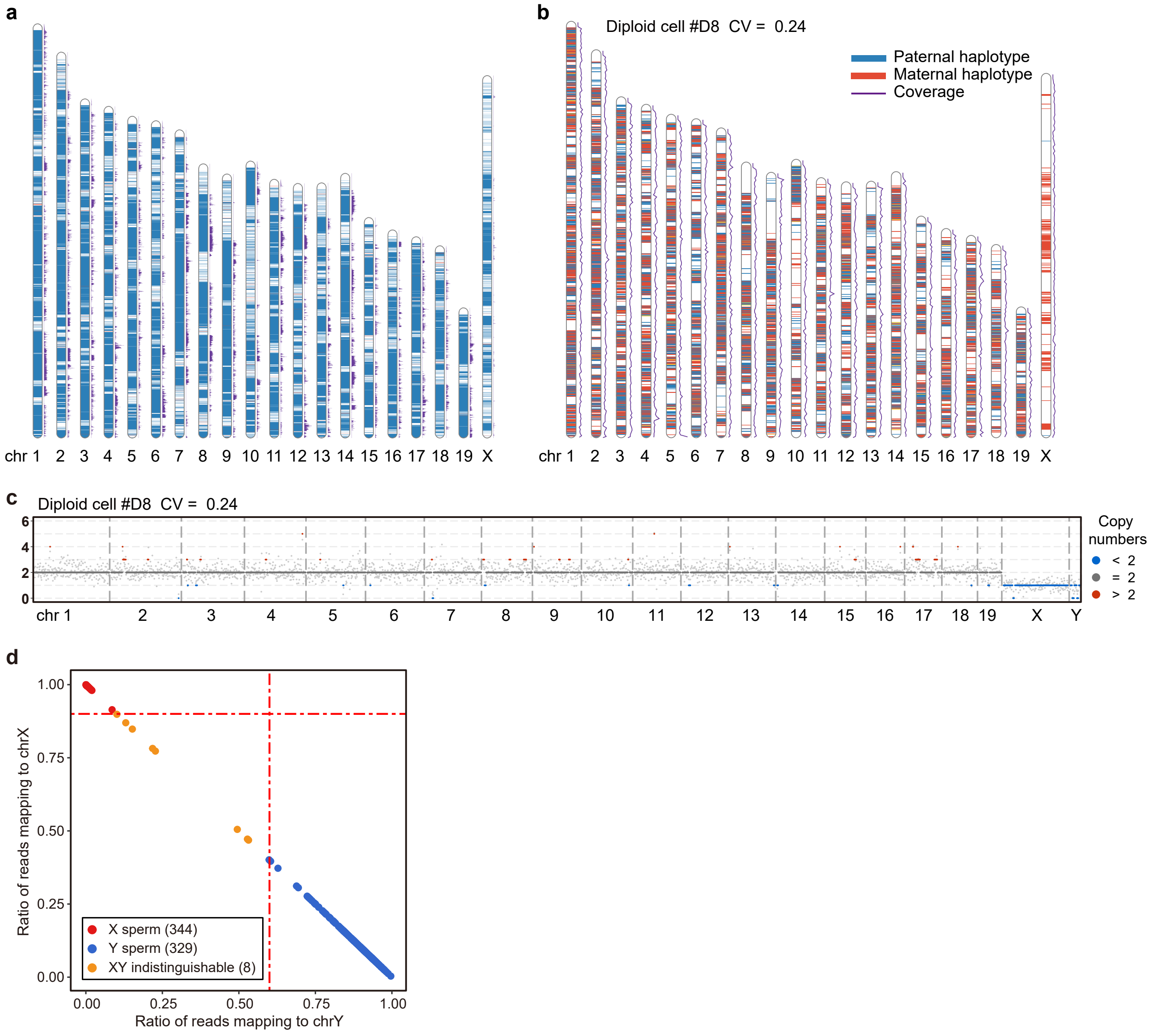

Supplementary Fig. S3 Distinction of X sperm cells and Y sperm cells and identification of contaminated diploid cells. a Distribution of benchmark set of hetSNPs in the chromosomes of B6D2F1 mice. **b** Parental haplotype contribution map from the diploid cell D8 on chromosome scale. **c** CNVs of the diploid cell D8, showing in 1 Mb windows. **d** Distinction of X sperm cells and Y sperm cells using ratio of reads mapping to X and Y chromosomes. 344 X sperm (red dots) and 329 Y sperm (blue dots) were identified, with 8 sperm being indistinguishable for X or Y (orange dots).

Supplementary Fig. S4

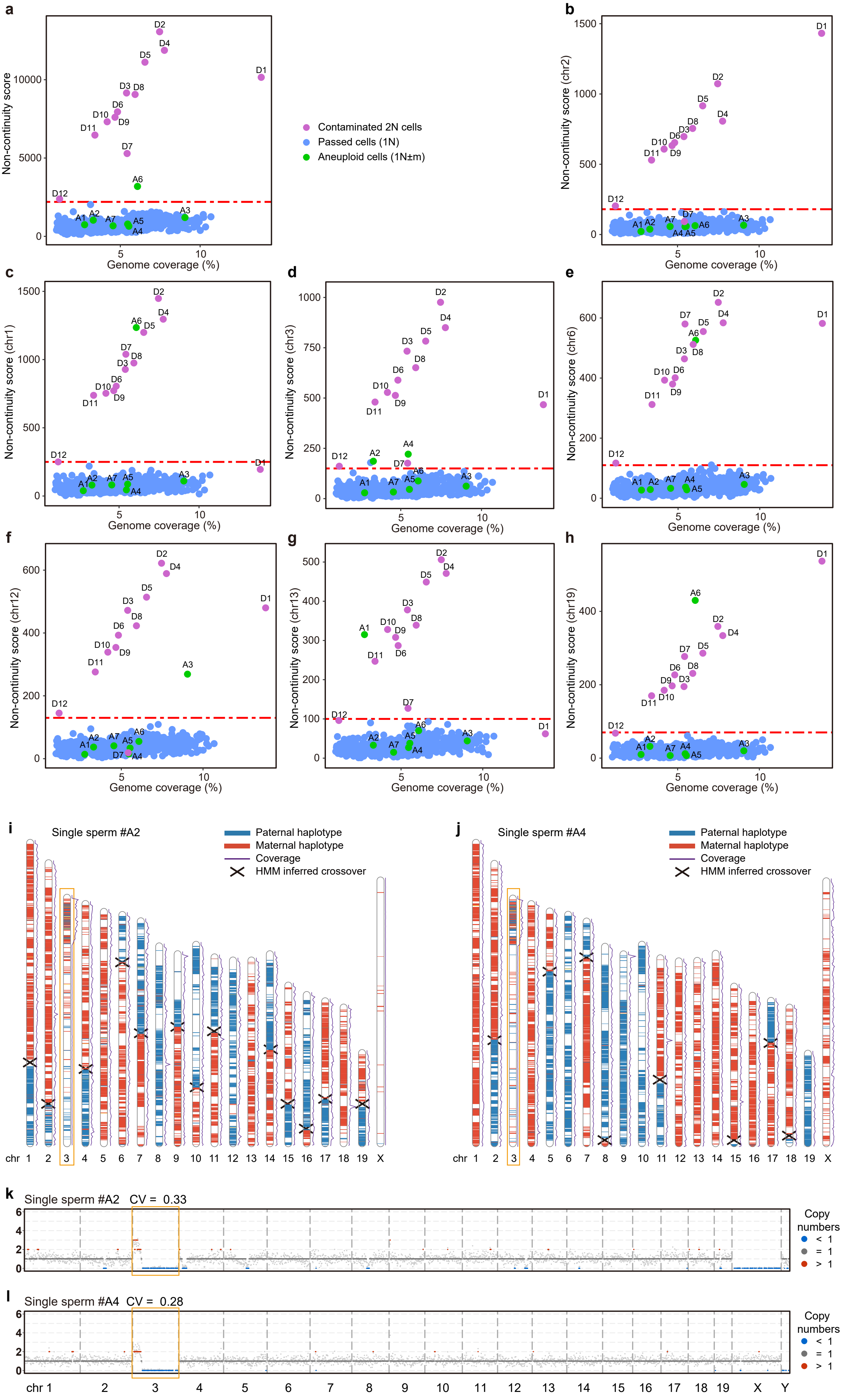

Supplementary Fig. S4 Identification of aneuploid sperm using 'non-continuity score'. a Non-continuity scores of all chromosomes in each sperm. Diploid cells are represented as purple dots and marked as D1-D12. Aneuploid sperm cells are represented as green dots and marked as A1-A7. **b**–**h** Non-continuity scores of specific chromosomes in each sperm. Diploid cells have much higher non-continuity scores across most chromosome (represented as purple dots) and aneuploid sperm have higher non-continuity scores across aneuploid chromosomes (represented as green dots). **i** Parental haplotype contribution map of the 20 chromosomes from the aneuploid sperm cell A2. Blue regions are the paternal SNPs and red regions are the maternal SNPs. Crossover sites are marked as forks. **j** Parental haplotype contribution map of the 20 chromosomes from the aneuploid sperm cell A4. Blue regions are the paternal SNPs and red regions are the maternal SNPs. Crossover sites are marked as forks. **k** CNVs of the aneuploid sperm A2, showing in 1 Mb windows. **l** CNVs of the aneuploid sperm A4, showing in 1 Mb windows.

Supplementary Fig. S5

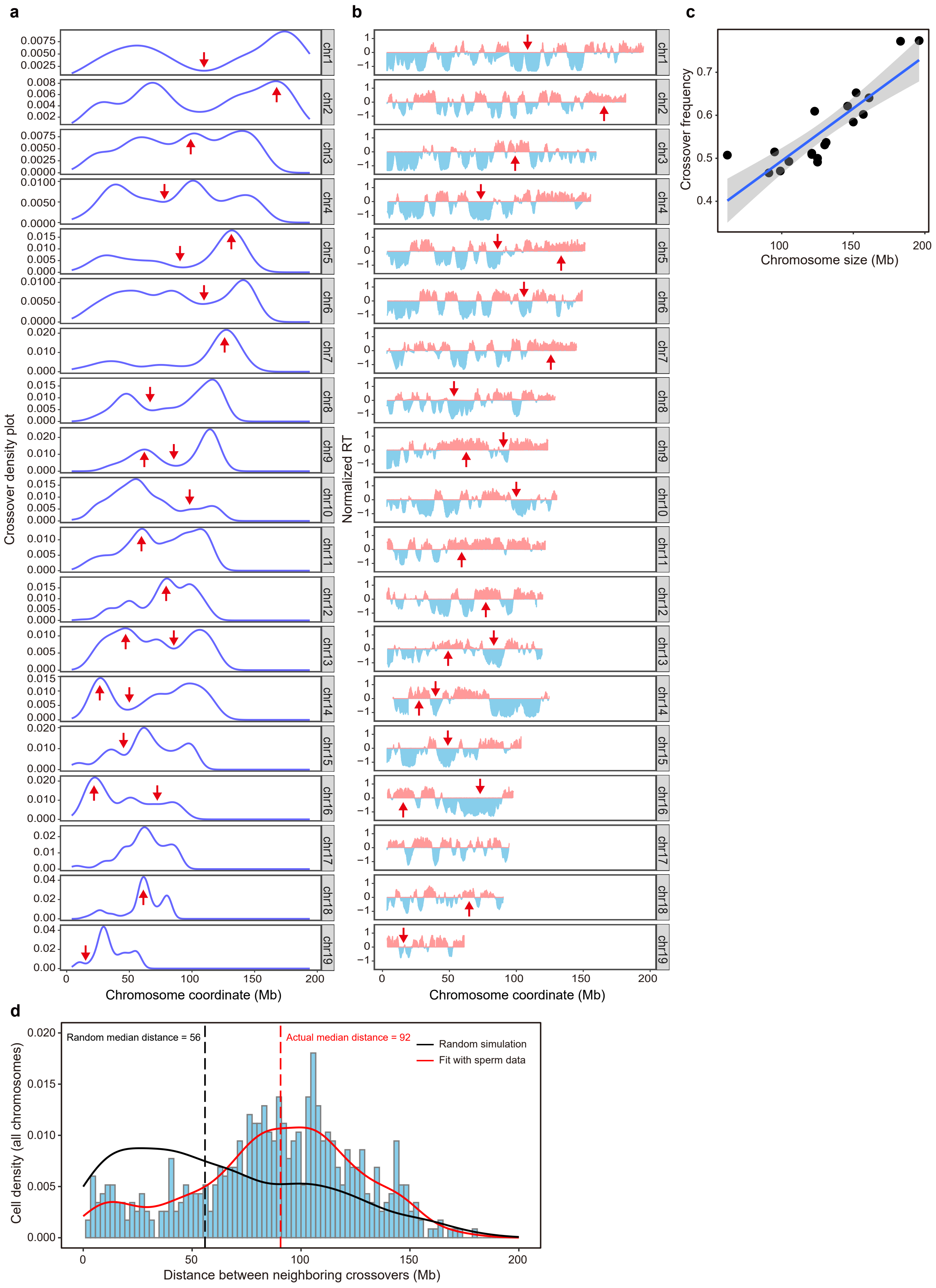

Supplementary Fig. S5 Distribution characteristics of crossovers in mouse sperm. a Crossover location density plots for each chromosome showing the crossover density by the distance from the centromere to telomere. Red arrows show regions corresponded with the position of DNA replication. **b** Replication timing (log2 of normalized sequencing coverage) in meiotic S-phase in testis from the previous study[32]. Replication timing is shown as filled pink (early replicating) and blue (late replicating) regions. Red arrows show regions corresponded with the crossover density. **c** Relationship between crossover frequency and chromosome size fitted with simple linear regression with a Pearson correlation of 0.86. Only autosomal whole-chromosome euploidies are included. **d** Distribution of inter-crossover distances measured in megabases of DNA length on all chromosomes. The red line represents the distribution curve fitting the experimental data. The black line shows the distribution of randomly generated distance. The dotted lines represent the median distances of the random distribution and experimental data distribution.

Supplementary Fig. S6

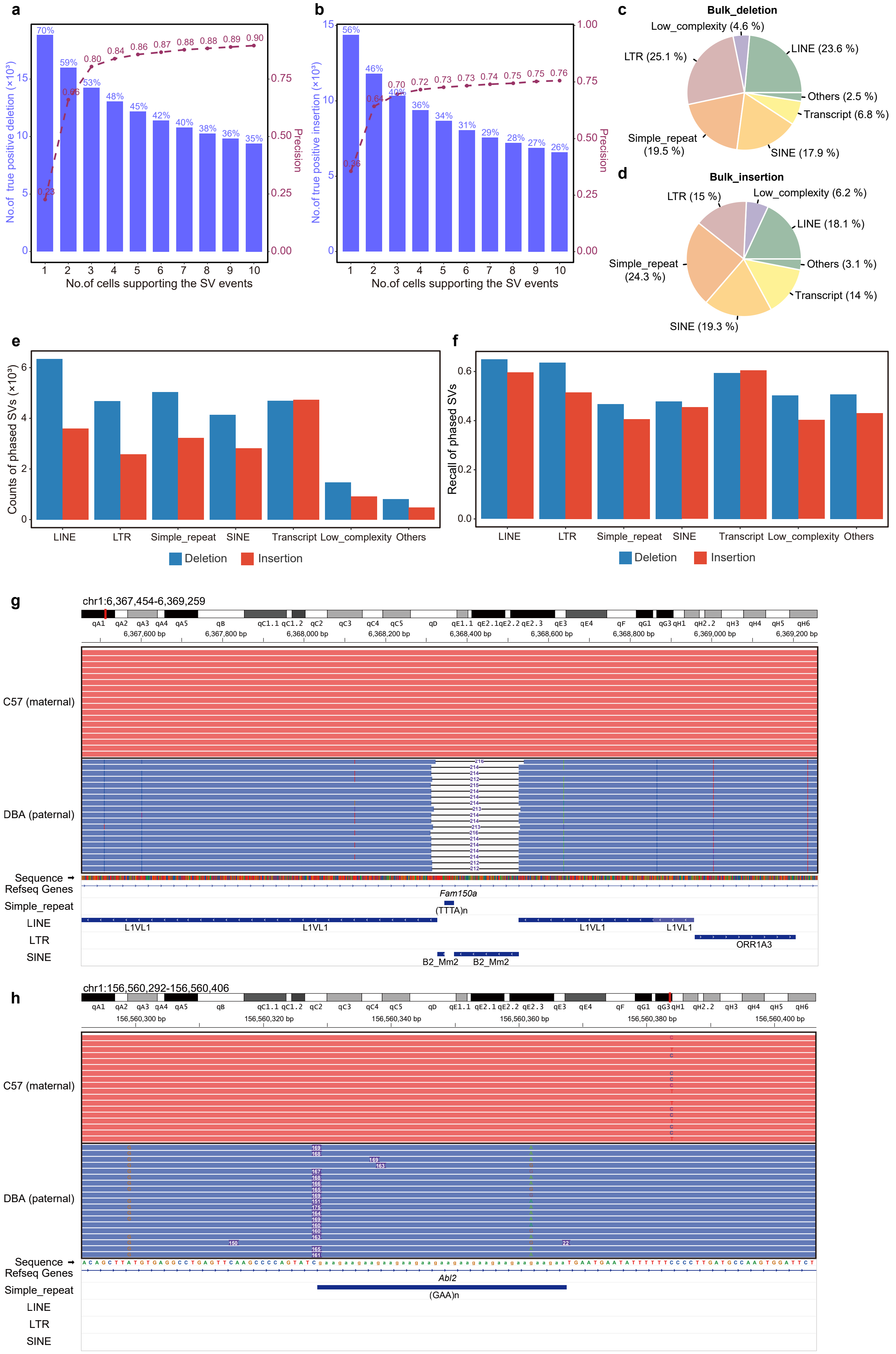

Supplementary Fig. S6 Detection and phasing of SVs in mouse sperm. a Precision of deletions detected by Refresh-seq and the percentage of true positive deletions with different numbers of supporting cells. **b** Precision of insertions detected by Refresh-seq and the percentage of true positive insertions with different numbers of supporting cells. **c** The proportion of different types of elements involved in the bulk benchmark deletion set. **d** The proportion of different types of elements involved in the bulk benchmark insertion set. **e** The counts of different types of elements involved in the successfully phased SVs. **f** The recall of different types of elements involved in phased SVs among the element annotation involved in bulk benchmark SVs set. **g** An Integrative Genomics Viewer illustration of a paternal (DBA) specific deletion of SINE element. **h** An Integrative Genomics Viewer illustration of a paternal (DBA) specific insertion near a single repeat region.

Supplementary Fig. S7

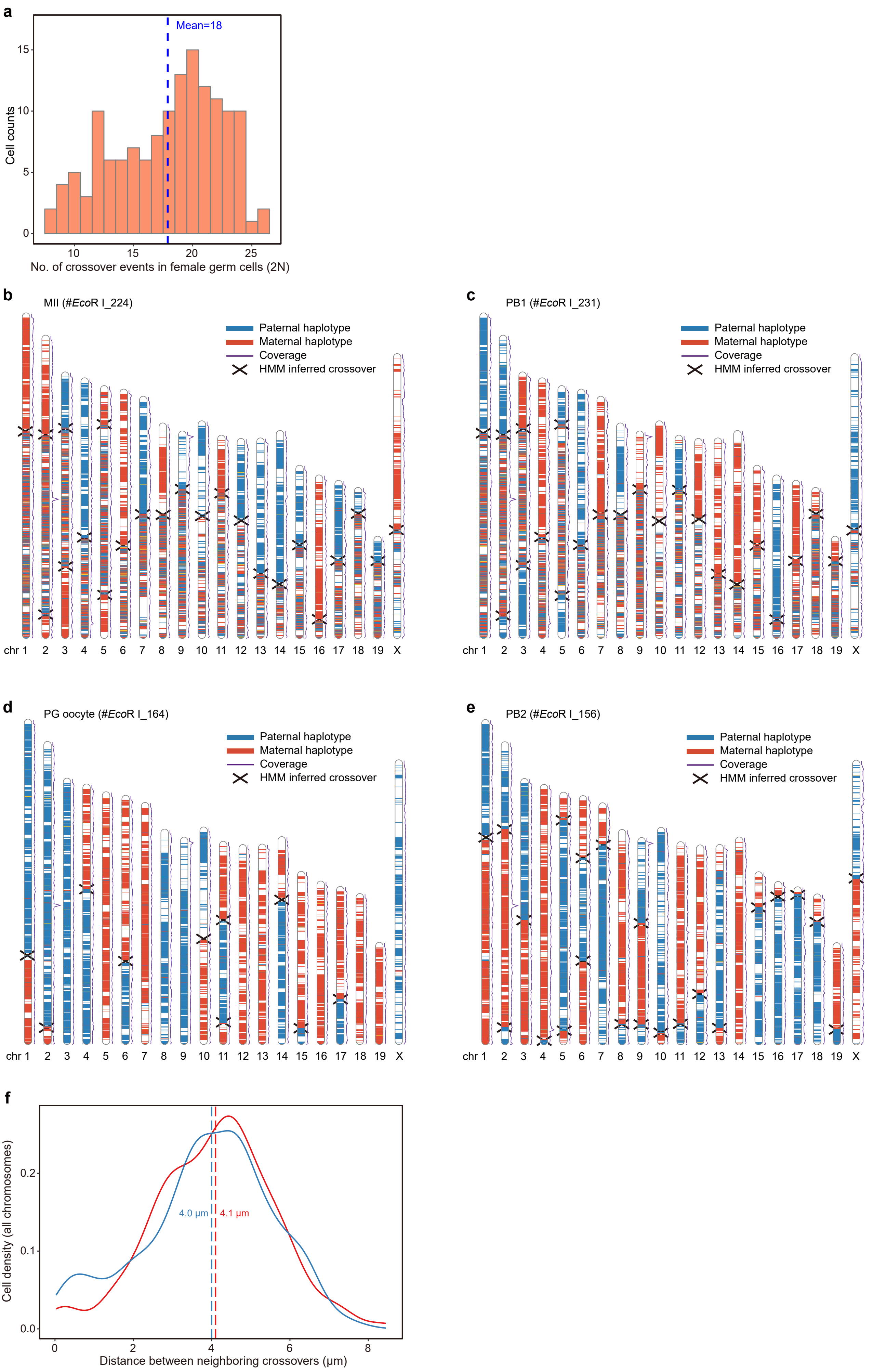

Supplementary Fig. S7 Parental haplotype contribution map of paired cells and recombination patterns in female mice. a Distribution of crossover number in diploid female cells, including PB1 and MII oocytes. **b** Parental haplotype contribution map of a MII oocyte. **c** Parental haplotype contribution map of a PB1 cell corresponded with the MII shown in Supplementary Fig. S7b. **d** Parental haplotype contribution map of a PG oocyte. **e** Parental haplotype contribution map of a PB2 cell corresponded with the PG oocyte shown in Supplementary Fig. S7d. **f** Distribution curves of inter-crossover distances on all chromosomes in female and male meiosis, measured on the physical distance scale (total synaptonemal complex length) from previous studies[68,69]. The dotted lines represent the median distances of the distribution curves. *P* value of Mann-Whitney U-test = 0.063.

Supplementary Fig. S8

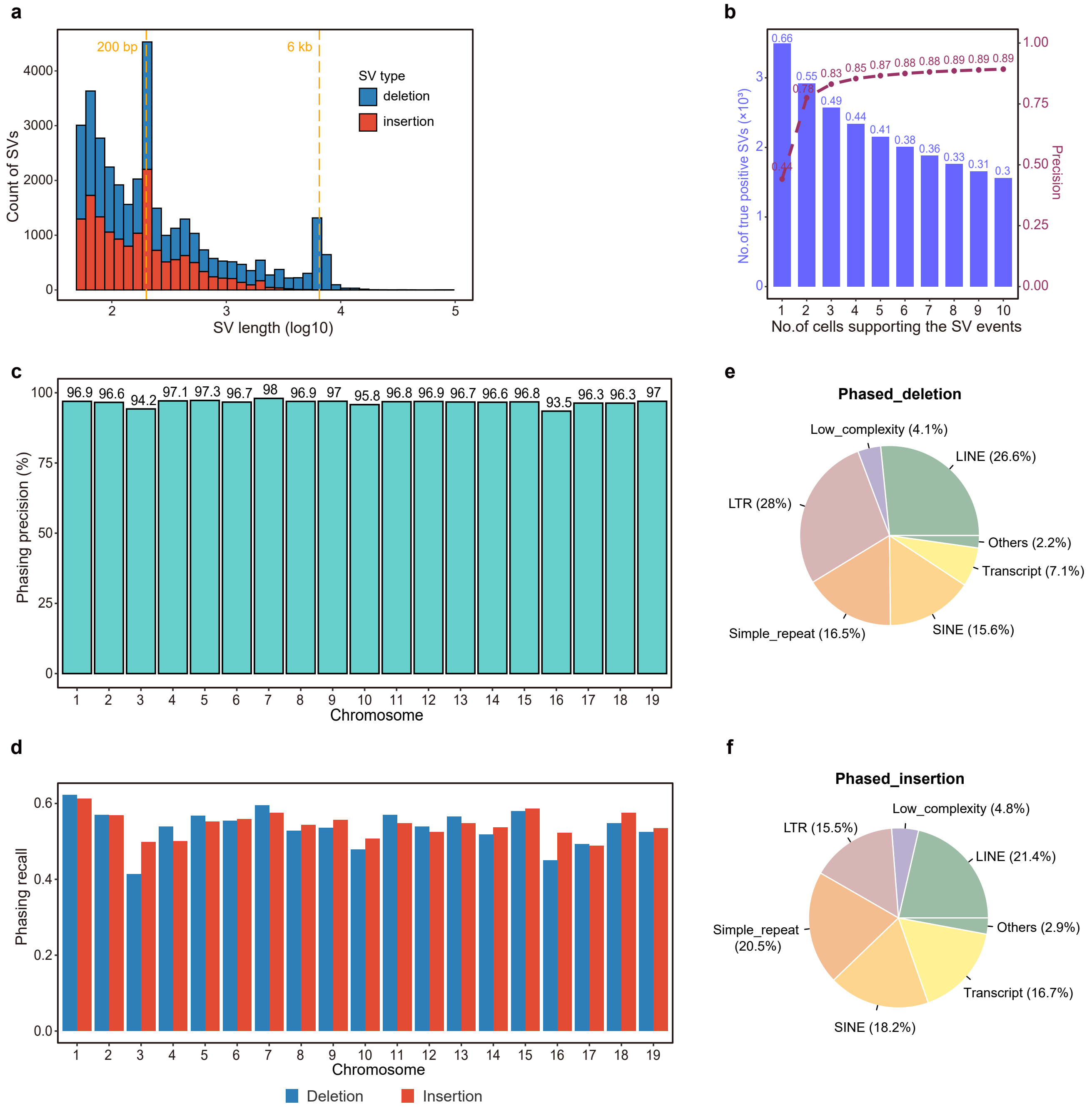

Supplementary Fig. S8 Detection and phasing of SVs in female mice. a Length distribution of identified true positive SVs in female. Deletions are shown in blue and insertions are shown in red. Local peaks of SV length are marked as orange dotted lines. **b** Precision of SVs (deletions and insertions) detected by Refresh-seq and the percentage of true positive SVs with different numbers of supporting haploid cells. **c** The genome-wide haplotype phasing precision of SVs on the chromosome scale. **d** The proportion of phased SVs in bulk benchmark SV set on the chromosome scale. Deletions are shown in blue and insertions are shown in red. **e** The proportion of different elements annotation involved in phased deletions. **f** The proportion of different elements annotation involved in phased insertions.

Supplementary Fig. S9

a

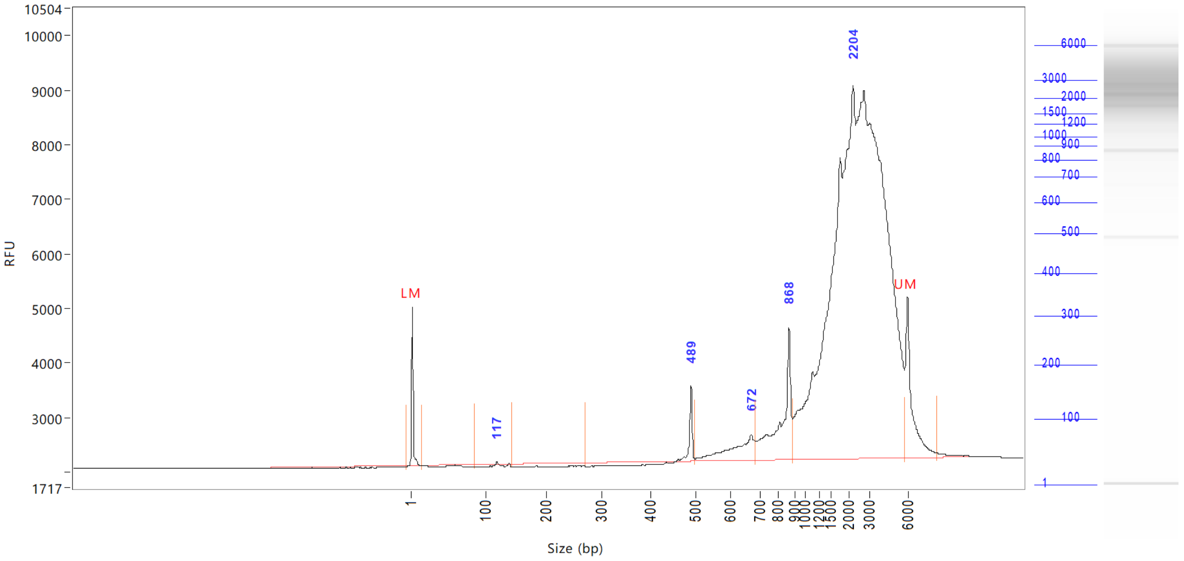

b

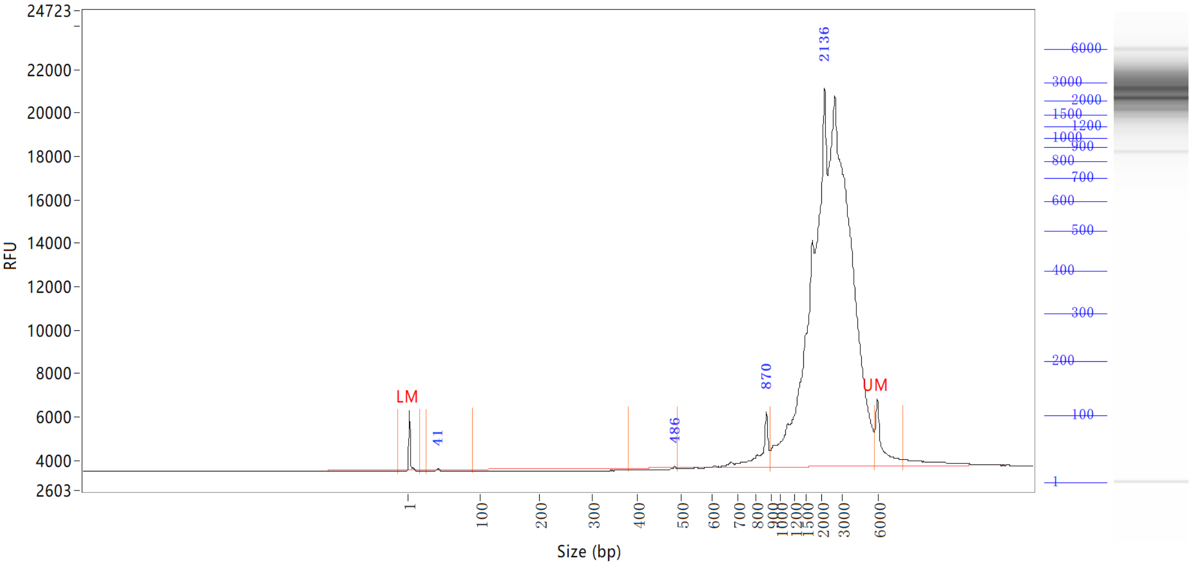

Supplementary Fig. S9 Typical library profiles of Refresh-seq. a Fragment analysis of library of Refresh-seq after 20 cycles of PCR program and purification. **b** Fragment analysis of library of Refresh-seq after the second round of amplification and purification.

Supplementary Methods

Protocol for Refresh-seq

Single cell collection and lysis

1. The lysis reaction was prepared each time before experiment following the recipe below.

Component	Final concentration	Per reaction (µL)
100 mM Tris-EDTA (1 M tris+0.1 M EDTA)	10 mM	0.25
Qiagen protease (20 mg/mL)	1 mg/mL	0.125
10% triton X-100	0.3%	0.075
1 M KCl	20 mM	0.05
H_2O	-	1.9625
1 M DTT	15 mM	0.0375
Total	-	2.5

2. Individual cells were collected with a microcapillary connected to a mouth pipette or by FACS after washed with 0.1% phosphate-buffered saline-bovine serum albumin for three times. Each single cell was placed into a 0.2-mL thin-walled PCR tube containing 2.5 µL lysis buffer.
3. The cells were lysed at 50 °C for 3 h to digest histones and then 70 °C for 30 min to inactivate the protease. After lysis, cells could be stored at -80 °C.

Restriction digestion of single cell gDNAs

4. Remove the lysis plate from -80 °C and thaw on ice.
5. Prepare the master mix for the restriction digestion reaction on ice as follows:

Component	Final concentration	Per reaction (µL)	Per 120 reactions (µL)
10× buffer	1×	0.5	60
Restriction Enzyme	1 unit	0.1	12
Water	-	1.9	228
Total	-	2.5	300

6. Split 36 µL of the master mix to each well of 8-strip tube, and then transfer 2.5 µL of the master mix to each of the sample wells by using the 8-channel pipette for a total liquid volume of 5 µL. Vortex and spin the tubes briefly.
7. Put the sample in the thermocycler with the lid temperature pre-set to 80 °C. Incubate the reaction to the recommended program according to the restriction enzyme used. For *Eco*R I and *Sac* I, the restriction digestion was performed at 37 °C 15 min, and then 65 °C 20 min to inactivate the restriction enzyme. For *Asi*S I, the restriction digestion was performed at 37 °C 1 h, and then 80 °C 20 min to inactivate the restriction enzyme.

   Program for *Eco*R I:

Order	Temperature (°C)	Duration (min)	Purpose
1	37	15	gDNA digestion
2	65	20	Inactivation of restriction enzymes
3	4	Hold	-

End repair and dA-tailing

8. This procedure produces end-repaired, 5'-phosphorylated, 3'-dA-tailed dsDNA fragments. Prepare the master mix as described in the following recipe on ice:

Component	Final concentration	Per reaction (µL)	Per 120 reactions (µL)
E&A buffer	1×	0.7	84
E&A Enzyme	-	0.3	36
Total	-	1	120

9. Split 14 µL of the master mix to each well of 8-strip tube, and then transfer 1 µL of the master mix to each of the sample wells by using the 8-channel pipette for a total liquid volume of 6 µL. Vortex and spin the tubes briefly.

10. Put the sample in the thermocycler with the lid temperature pre-set to 80 °C. Incubate the reaction according to the program below:

Order	Temperature (°C)	Duration (min)	Purpose
1	20	30	Filling in cut sites and A-tailing
2	65	30	Inactivation of E&A Enzyme
3	4	Hold	-

Adapter ligation

11. During this step, dsDNA adapters with 3'-dTMP overhangs are ligated to 3'-dA-tailed molecules. Dilute adapter (NEBNext Singleplex Oligos for Illumina) stocks as 1:10, to a

concentration of 2 µM. Prepare the master mix as described in the following recipe on ice:

Component	Final concentration	Per reaction (µL)	Per 120 reactions (µL)
Ligation buffer	1×	3	360
Ligase	-	1	120
Adaptor (1:10)	200 nM	0.5	60
H_2O	-	0.5	60
Total	-	5	600

12. Split 74 µL of the master mix to each well of 8-strip tube, and then transfer 5 µL of the master mix to each of the sample wells by using the 8-channel pipette for a total liquid volume of 11 µL. Vortex and spin the tubes briefly.
13. Put the sample in the thermocycler and incubate the ligation reaction at 20 °C for 30 min in the thermocycler with the heated lid off.
14. Add 0.5 µL USER (uracil-specific excision reagent) Enzyme to the ligation mixture, resulting in total volume of 11.5 µL. Mix well and incubate at 37 °C for 30 minutes with the heated lid set to 45 °C. Proceed immediately to purification of the ligation reaction.

DNA purification by using Agencourt AMPure beads

15. Take AMPure XP beads from the refrigerator and leave at room temperatures for at least 20 min. Gently shake the AMPure XP bottle to resuspend any magnetic particles that may have settled. Transfer 200 µL of AMPure XP beads to each well of 8-strip tube and then use an 8-channel pipette to dispense 11.5 µL (1×) of the beads to each sample well and

pipette up and down to mix thoroughly.

16. Let the mixed samples incubate at least 8 min at room temperature to bind PCR products to paramagnetic beads.
17. Place the reaction samples onto a magnetic grate and wait for the solution to clear, allowing beads to accumulate on one side of the well before proceeding to the next step.
18. Carefully take the supernatant and discard it by using a 100-µL 8-channel pipette.
19. Dispense 100 µL of freshly prepared 80% (vol/vol) ethanol to each well without disturbing the beads and then aspirate the ethanol and discard it. Repeat the ethanol wash once. If it remains ethanol in the sample wells, 10-µL pipette can be used to discard it.
20. Let the sample air-dry until there is no apparent ethanol residual on the beads' surface. Caution: over-drying the beads may result in reduced yield.
21. Remove the tubes from the magnet. Add 12 µL PCR graded water to the bead side and let the water run down through the beads. Pipette up and down to resuspend the beads thoroughly.
22. Let the samples incubate at least 5 min at room temperature to elute dsDNA from paramagnetic beads thoroughly.
23. Place the samples onto a magnetic grate and wait for the solution to clear. Carefully take 10 µL of the supernatant to new 8-strip tubes.

Library Amplification

24. Assemble each library amplification reaction as follows:

Component	Final concentration	Per reaction (μL)	Per 120 reactions (μL)
2× Gflex PCR Buffer (Mg^{2+}, dNTP plus)	1×	12.5	1500
Tks Gflex DNA Polymerase (1.25 U/ μL)	0.625 U	0.5	60
Barcode-P5	0.4 μM	1	Add to each well
Barcode-P3	0.4 μM	1	Add to each well
Total	-	15	

25. Split 190 μL of the master mix (2 × Gflex PCR Buffer mixed with Tks Gflex DNA Polymerase) to each well of 8-strip tube, and then transfer 13 μL of the master mix to each of the sample wells by using the 8-channel pipette. Add 2 μL pre-mixed barcode (Barcode-P5 and Barcode-P3 mixed with equal volume) to each sample well for a total liquid volume of 25 μL and make sure each well has a different combination of barcode P3 (ATCG-[24 bp P3-barcode 1-24]-GACTGGAGTTCAGACGTGTGCT) and barcode P5 (GCTA-[24 bp P5-barcode 81-96]-TACACTCTTTCCCTACACGACGCTCTTCCGATCT). Mix and spin down the tubes briefly.
26. Put the sample in the thermocycler with the lid temperature pre-set to 105 °C. Incubate the reaction according to the program below:

Cycle number	Denature	Anneal	Extend	Hold
1	98 °C, 45 s	-	-	-
2-22	98 °C, 15 s	65 °C, 30 s	72 °C, 5 min	-
23	-	-	72 °C, 5 min	-
24	-	-	-	4 °C

Purification of PCR products

27. Repeat Steps 15–23, but use only 17.5 µL AMPure XP beads in a ratio of 0.7:1 (0.7×) and elute with 20 µL PCR graded water. Then repeat Steps 15–23 again, and use 14 µL AMPure XP beads (0.7×) and elute with 10 µL PCR graded water. This will minimize the carryover of primer dimers.

28. Measure the concentration of each sample well by Qubit according to the manufacturer's instructions. Check the size distribution on high sensitivity DNA analysis such as the Agilent 5200 Fragment Analyzer system. Size distribution varies according to the restrict enzyme used in the digestion. For *EcoRI*, a peak with an average size of 1000-3000 bp will be observed (Supplementary Fig. S9a).

Second round of amplification

29. If the concentrations of the samples are too low for sequencing, the second round of amplification can be applied to the purified samples. Repeat Steps 24–28, but only purify the products with 15 µL AMPure XP beads (0.6×) and elute with 20 µL PCR graded water. Then repeat Steps 15–23 again, and use 12 µL AMPure XP beads (0.6×) and elute with 10 µL PCR graded water.

30. For *Eco*R I, a narrower peak with an average size of 1000-3000 bp will be observed after

additional amplification and purification (Supplementary Fig. S9b).

DNA sequencing

31. Samples with different barcode can be pooled together according to the raw data needed. For example, if cell A is to be sequenced to 1 Gb reads, 10 ng of this sample should be taken. Proportionally, cell B wanted to be sequenced to 2 Gb reads should be taken 20 ng. A total of 1 μg DNA can be sequenced with a nanopore flow cell to generate 100 Gb of data.

Protocol for Refresh-seq (multiplexed)

For multiplexed version of this method, barcoded adaptors were used to increase experimental throughput. In brief, after end repair and dA-tailing, single-cell gDNA fragments were ligated with barcoded adaptors prepared ahead. Cells with different barcodes were pooled together and purified with Agencourt AMPure beads, after which library amplification was performed to generate enough DNA for sequencing.

Preparation of barcoded adaptors

A. Single-stranded oligos need to be annealed with their appropriate partner before ligation. Synthesized oligos (NEB same-A: GATCGGAAGAGCACACGTCTGAACTCCAGTC with 5'Phospholation modification and Barcoded-B: ACACTCTTTCCCTACACGAC-[24 bp adaptor-barcode 31-46]-GCTCTTCCGATC*T) are dissolved to an initial concentration of 100 µM. To create a duplex adapter, combine NEB same-A and Barcoded-B in a 1:1 ratio for a total annealed adapter concentration of 50 µM (use 50 µL adapter NEB same-A and 50 µL adapter Barcoded-B) and mix thoroughly.

B. Put the sample in the thermocycler with the lid temperature pre-set to 99 °C. Incubate the reaction according to the program below:

Loops	Step	Block temperature (°C)	Hold time (sec)	Go to	Cycles	Temperature increment (ΔT) (°C)	Ramp rate (ΔR) (°C/s)
	1	95	12	-	-	-	6
100×	2	95	2	2	100	-0.1	6
100×	3	85	4	3	100	-0.1	6
700×	4	75	10	4	700	-0.1	6
	5	5	∞	-	-	-	6

C. Dilute the annealed adapters to a final working strength concentration of 2 µM and store at -20 °C. For convenience, it is also possible to store the adapters at 4 °C while in active use.

D. Perform single cell lysis, restriction enzyme digestion and end repair and dA-tailing as previously described (Steps 1–10).

E. Prepare the master mix as described in the following recipe on ice:

Component	Final concentration	Per reaction (µL)	Per 120 reactions (µL)
Ligation buffer	1×	3	360
Ligase	-	1	120
H2O	-	0.5	60
Total	-	4.5	540

F. Split 63 µL of the master mix to each well of 8-strip tube. Add 7 µL barcoded adaptor (from step C) to each well and make sure the mixture in 8 wells features different barcode.

Pipette up and down to mix thoroughly and transfer 5 µL of the master mix to each of the sample wells by using the 8-channel pipette for a total liquid volume of 11 µL. Vortex and spin the tubes briefly.

G. Put the sample in the thermocycler and incubate the ligation reaction at 20 °C for 30 min in the thermocycler with the heated lid off. USER enzyme step is eliminated as "Y" shaped adaptors have been directly annealed and deoxyU is no longer needed.

H. Pool 8 cells with different barcode (usually in a row) together, purify the mixed products with 80 µL AMPure XP beads (1×) and elute with 20 µL PCR graded water.

I. Assemble each library amplification reaction as follows:

Component	Final concentration	Per reaction (µL)
2× Gflex PCR Buffer (Mg^{2+}, dNTP plus)	1×	25
Tks Gflex DNA Polymerase (1.25 U/ µL)	1.25 U	1
Common-P5	0.4 µM	2
Barcode-P3	0.4 µM	2
Total	-	30

J. Split 26 µL of the master mix (2× Gflex PCR Buffer mixed with Tks Gflex DNA Polymerase) to each sample well, and add 4 µL pre-mixed barcode (Common-P5 and Barcode-P3 mixed with equal volume) to each well for a total liquid volume of 50 µL. Make sure each well has a different barcode-P3 (ATCG-[24 bp P3-barcode 1-24]-GACTGGAGTTCAGACGTGTGCT). Mix and spin down the tubes briefly.

K. Perform library amplification and purification as previously described (step 26-28) but only use 35 µL AMPure XP beads in a ratio of 0.7:1 (0.7×) and elute with 20 µL PCR graded water in step 27. The second round of amplification can also be performed if needed.

Supplementary Table S1. Accuracy of haplotype phasing of SNPs and SVs

Type of genomic variants	SNPs	SVs	SVs
**Gender**	Male	Male	Female
**Sample**	Sperm	Sperm	PB2 and PG oocytes
**Cell number**	673	673	255
**No. of heterozygous genomic variants in B6D2F1 mice**	4,343,205	54,471	54,471
**No. of phased genomic variants**	1,983,794 (45.68%)	27,490 (50.47%)	29,429 (54.03%)
**No. of phased genomic variants consistent with gold standard**	1,983,152 (99.97%)	27,172 (98.84%)	28,444 (96.65%)
**The length of phased blocks**	Whole chromosome	Whole chromosome	Whole chromosome

Supplementary Table S2. Primers for Refresh-seq

**Barcode-P3**	Sequence (5' - 3')
P3-bar1	ATCGAAGAAAGTTGTCGGTGTCTTTGTGGACTGGAGTTCAGACGTGTGCT
P3-bar2	ATCGTCGATTCCGTTTGTAGTCGTCTGTGACTGGAGTTCAGACGTGTGCT
P3-bar3	ATCGGAGTCTTGTGTCCCAGTTACCAGGGACTGGAGTTCAGACGTGTGCT
P3-bar4	ATCGTTCGGATTCTATCGTGTTTCCCTAGACTGGAGTTCAGACGTGTGCT
P3-bar5	ATCGCTTGTCCAGGGTTTGTGTAACCTTGACTGGAGTTCAGACGTGTGCT
P3-bar6	ATCGTTCTCGCAAAGGCAGAAAGTAGTCGACTGGAGTTCAGACGTGTGCT
P3-bar7	ATCGGTGTTACCGTGGGAATGAATCCTTGACTGGAGTTCAGACGTGTGCT
P3-bar8	ATCGTTCAGGGAACAAACCAAGTTACGTGACTGGAGTTCAGACGTGTGCT
P3-bar9	ATCGAACTAGGCACAGCGAGTCTTGGTTGACTGGAGTTCAGACGTGTGCT
P3-bar10	ATCGAAGCGTTGAAACCTTTGTCCTCTCGACTGGAGTTCAGACGTGTGCT
P3-bar11	ATCGGTTTCATCTATCGGAGGGAATGGAGACTGGAGTTCAGACGTGTGCT
P3-bar12	ATCGCAGGTAGAAAGAAGCAGAATCGGAGACTGGAGTTCAGACGTGTGCT
P3-bar13	ATCGAGAACGACTTCCATACTCGTGTGAGACTGGAGTTCAGACGTGTGCT
P3-bar14	ATCGAACGAGTCTCTTGGGACCCATAGAGACTGGAGTTCAGACGTGTGCT
P3-bar15	ATCGAGGTCTACCTCGCTAACACCACTGGACTGGAGTTCAGACGTGTGCT
P3-bar16	ATCGCGTCAACTGACAGTGGTTCGTACTGACTGGAGTTCAGACGTGTGCT
P3-bar17	ATCGACCCTCCAGGAAAGTACCTCTGATGACTGGAGTTCAGACGTGTGCT
P3-bar18	ATCGCCAAACCCAACAACCTAGATAGGCGACTGGAGTTCAGACGTGTGCT
P3-bar19	ATCGGTTCCTCGTGCAGTGTCAAGAGATGACTGGAGTTCAGACGTGTGCT
P3-bar20	ATCGTTGCGTCCTGTTACGAGAACTCATGACTGGAGTTCAGACGTGTGCT
P3-bar21	ATCGGAGCCTCTCATTGTCCGTTCTCTAGACTGGAGTTCAGACGTGTGCT
P3-bar22	ATCGACCACTGCCATGTATCAAAGTACGGACTGGAGTTCAGACGTGTGCT
P3-bar23	ATCGCTTACTACCCAGTGAACCTCCTCGGACTGGAGTTCAGACGTGTGCT
P3-bar24	ATCGGCATAGTTCTGCATGATGGGTTAGGACTGGAGTTCAGACGTGTGCT
**Barcode-P5**	Sequence (5' - 3')
P5-bar96	GCTACTGAACGGTCATAGAGTCCACCATTACACTCTTTCCCTACACGACGCTCTTCCGATCT
P5-bar95	GCTACCTGTCTGGAAGAAGAATGGACTTTACACTCTTTCCCTACACGACGCTCTTCCGATCT
P5-bar94	GCTAGATTGTCCTCAAACTGCCACCTACTACACTCTTTCCCTACACGACGCTCTTCCGATCT
P5-bar93	GCTAAGTTTCCATCACTTCAGACTTGGGTACACTCTTTCCCTACACGACGCTCTTCCGATCT
P5-bar92	GCTATTGTGAGTGGAAAGATACAGGACCTACACTCTTTCCCTACACGACGCTCTTCCGATCT
P5-bar91	GCTAGGCTCCATAGGAACTCACGCTACTTACACTCTTTCCCTACACGACGCTCTTCCGATCT
P5-bar90	GCTAGATGTAGAGGGTACGGTTTGAGGCTACACTCTTTCCCTACACGACGCTCTTCCGATCT

P5-bar89	GCTAACAGCATCAATGTTTGGCTAGTTGTACACTCTTTCCCTACACGACGCTCTTCCGATCT
P5-bar88	GCTATCTTCTACTACCGATCCGAAGCAGTACACTCTTTCCCTACACGACGCTCTTCCGATCT
P5-bar87	GCTATACATGCTCCTGTTGTTAGGGAGGTACACTCTTTCCCTACACGACGCTCTTCCGATCT
P5-bar86	GCTAAGGTGATCCCAACAAGCGTAAGTATACACTCTTTCCCTACACGACGCTCTTCCGATCT
P5-bar85	GCTAAACGGAGGAGTTAGTTGGATGATCTACACTCTTTCCCTACACGACGCTCTTCCGATCT
P5-bar84	GCTAGTAGTGGACCTAGAACCTGTGCCATACACTCTTTCCCTACACGACGCTCTTCCGATCT
P5-bar83	GCTATGGCTTGATCTAGGTAAGGTCGAATACACTCTTTCCCTACACGACGCTCTTCCGATCT
P5-bar82	GCTAACGGTATGTCGAGTTCCAGGACTATACACTCTTTCCCTACACGACGCTCTTCCGATCT
P5-bar81	GCTACCTCATCTTGTGAAGTTGTTTCGGTACACTCTTTCCCTACACGACGCTCTTCCGATCT
**Barcoded-B**	Sequence (5’ - 3’)
Bar31-B	ACACTCTTTCCCTACACGACTCAGTGAGGATCTACTTCGACCCAGCTCTTCCGATC*T
Bar32-B	ACACTCTTTCCCTACACGACCCAGTAGAAGTCCGACAACGTCATGCTCTTCCGATC*T
Bar33-B	ACACTCTTTCCCTACACGACCAGACTTGGTACGGTTGGGTAACTGCTCTTCCGATC*T
Bar34-B	ACACTCTTTCCCTACACGACGGACGAAGAACTCAAGTCAAAGGCGCTCTTCCGATC*T
Bar35-B	ACACTCTTTCCCTACACGACCTACTTACGAAGCTGAGGGACTGCGCTCTTCCGATC*T
Bar36-B	ACACTCTTTCCCTACACGACATGTCCCAGTTAGAGGAGGAAACAGCTCTTCCGATC*T
Bar37-B	ACACTCTTTCCCTACACGACGCTTGCGATTGATGCTTAGTATCAGCTCTTCCGATC*T
Bar38-B	ACACTCTTTCCCTACACGACACCACAGGAGGACGATACAGAGAAGCTCTTCCGATC*T
Bar39-B	ACACTCTTTCCCTACACGACCCACAGTGTCAACTAGAGCCTCTCGCTCTTCCGATC*T
Bar40-B	ACACTCTTTCCCTACACGACTAGTTTGGATGACCAAGGATAGCCGCTCTTCCGATC*T
Bar41-B	ACACTCTTTCCCTACACGACGGAGTTCGTCCAGAGAAGTACACGGCTCTTCCGATC*T
Bar42-B	ACACTCTTTCCCTACACGACCTACGTGTAAGGCATACCTGCCAGGCTCTTCCGATC*T
Bar43-B	ACACTCTTTCCCTACACGACCTTTCGTTGTTGACTCGACGGTAGGCTCTTCCGATC*T
Bar44-B	ACACTCTTTCCCTACACGACAGTAGAAAGGGTTCCTTCCCACTCGCTCTTCCGATC*T
Bar45-B	ACACTCTTTCCCTACACGACGATCCAACAGAGATGCCTTCAGTGGCTCTTCCGATC*T
Bar46-B	ACACTCTTTCCCTACACGACGCTGTGTTCCACTTCATTCTCCTGGCTCTTCCGATC*T
**Other**	Sequence (5’ - 3’)
NEB same-A	5'-phos-GATCGGAAGAGCACACGTCTGAACTCCAGTC
Common-P5	ACACTCTTTCCCTACACGAC

* represents phosphorothioate bond.
